# Synthesis and Characterization of Bipyridyl-(Imidazole)_n_ Mn(II) Compounds and Their Evaluation as Potential Precatalysts for Water Oxidation

**DOI:** 10.3390/molecules28207221

**Published:** 2023-10-23

**Authors:** Ge Mu, Ryan B. Gaynor, Baylee N. McIntyre, Bruno Donnadieu, Sidney E. Creutz

**Affiliations:** Department of Chemistry, Mississippi State University, Mississippi State, Starkville, MS 39762, USA; mugesysu@gmail.com (G.M.); rbg123@msstate.edu (R.B.G.); baylee.n.mcintyre@vanderbilt.edu (B.N.M.); bdonnadieu@chemistry.msstate.edu (B.D.)

**Keywords:** manganese, bioinorganic chemistry, coordination chemistry

## Abstract

Metalloenzymes make extensive use of manganese centers for oxidative catalysis, including water oxidation; the need to develop improved synthetic catalysts for these processes has long motivated the development of bioinspired manganese complexes. Herein, we report a series of bpy-(imidazole)_n_ (n = 1 or 2) (bpy = 2,2′-bipyridyl) ligands and their Mn^2+^ complexes. Four Mn^2+^ complexes are structurally characterized using single-crystal X-ray diffraction, revealing different tridentate and tetradentate ligand coordination modes. Cyclic voltammetry of the complexes is consistent with ligand-centered reductions and metal-centered oxidations, and UV-vis spectroscopy complemented by TD-DFT calculations shows primarily ligand-centered transitions with minor contributions from charge-transfer type transitions at higher energies. In solution, ESI-MS studies provide evidence for ligand reorganization, suggesting complex speciation behavior. The oxidation of the complexes in the presence of water is probed using cyclic voltammetry, but the low stability of the complexes in aqueous solution leads to decomposition and precludes their ultimate application as aqueous electrocatalysts. Possible reasons for the low stability and suggestions for improvement are discussed.

## 1. Introduction

Manganese ions play central roles in a wide range of metalloenzymes essential to life. Due to the rich oxidation state landscape commonly available to them (ranging at least from Mn^2+^ to Mn^4+^ in biological systems and beyond that in synthetic systems), manganese centers feature prominently in many enzymes that catalyze redox processes, including key small-molecule transformations such as water oxidation and superoxide disproportionation. Motivated in part by these examples in Nature, there has been a long-standing interest in the development of synthetic manganese complexes and catalysts that mimic the structure and/or function of manganese-containing metalloenzymes, particularly for oxidative transformations.

**Figure 1:**
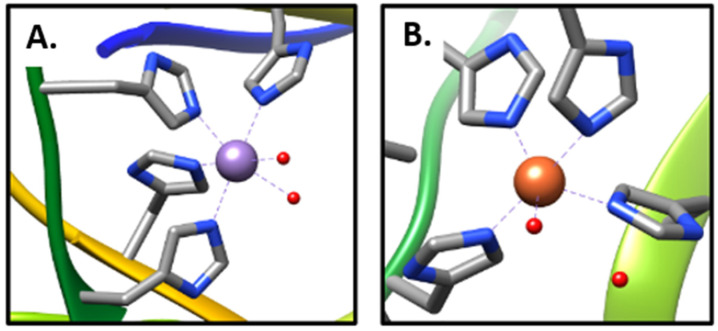
(**A**) Depiction of the manganese binding site in the TM1459 cupin enzyme. (**B**) Depiction of the iron-binding active site in the apocarotenoid-15,15′-oxygenase enzyme. (Blue = nitrogen; gray = carbon; red = oxygen; purple = manganese; orange = iron).

Manganese centers in nature, especially those in monometallic or dimetallic active sites, are frequently supported by one or more histidine ligands. Coordination environments featuring three histidine ligands and one carboxylate (e.g., aspartate) coordinated to a manganese ion feature in a number of well-studied monometallic manganese enzymes, including superoxide dismutase and oxalate oxidase [1,2]. Examples of structurally characterized proteins containing a manganese center coordinated to more than three histidine ligands are uncommon but include some examples in proteins with particularly intriguing reactivity. For example, the cupin protein TM1459 has been determined to contain a tetrahistidine-coordinated manganese ion (Figure 1A); in vitro, this protein catalyzes oxidative alkene cleavage of styrene derivatives when combined with *t*-BuOOH, although the in vivo role of this protein is not currently known [3,4]. The coordination environment of manganese in this protein—four histidines, no other protein-derived ligands, and two cis-disposed labile sites which are occupied by water-derived ligands in the crystal structure—seems to be unique among crystallographically characterized manganese metalloenzymes. Interestingly, an analogous coordination environment is present in the iron-containing metalloenzyme apocarotenoid-15,15′-oxygenase, which also catalyzes oxidative alkene cleavage in vivo [5] (Figure 1B). Taken together, these facts may suggest that this type of coordination environment and geometry is well-suited to oxidative cleavage reactivity. Mechanistic studies on manganese model complexes have also suggested that the presence of cis-disposed vacant sites is important for other types of oxidative reactivity, including the oxidation of water to O_2_ [6,7,8,9].

**Figure 2:**
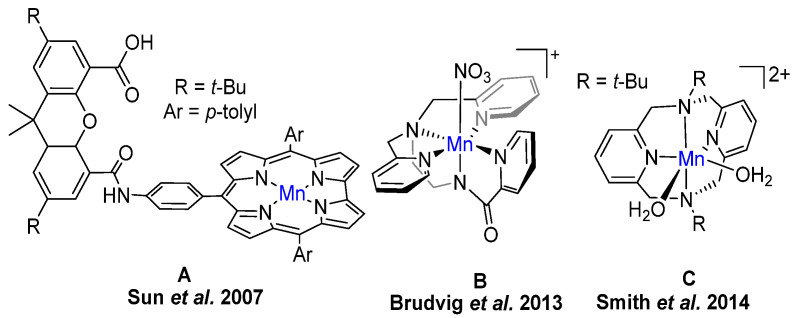
Selection of reported mononuclear manganese complexes that mediate or catalyze water oxidation [17,22,23].

Water oxidation has frequently been a reaction of interest for manganese chemistry due to the central role of manganese in the Mn_4_CaO_5_ cluster featured in the active site of the oxygen-evolving cluster [10]. Because of the complexity of this active site, synthetic mimics have often focused on simplified systems. Some of the most successful functional mimics of manganese in the OEC have been dinuclear Mn complexes containing µ-oxo bridges, supported by multidentate nitrogen-based ligand frameworks such as terpyridines; mechanistic studies on these systems have often highlighted the important role of manganese oxo or oxyl intermediates [11,12,13,14,15]. Efficient cluster-based mimics have also been reported [16]. Compared with the dinuclear µ-oxo compounds, only a few mononuclear manganese complexes have been reported to mediate or catalyze water oxidation; Figure 2 shows some notable examples. A xanthene-appended manganese corrole complex (Figure 2A) reported by Sun was one of the first mononuclear compounds reported to be able to mediate water oxidation electrochemically, although the reaction was substoichiometric (turnover number (TON) = 0.05) [17]. The success of several related manganese corrole complexes in water oxidation reactions has been attributed in part to their ability to stabilize the Mn(III) resting state and promote further oxidation to higher oxidation states, including manganese oxos, at relatively mild redox potentials [17,18,19,20,21]. A mononuclear system reported by the Brudvig group (Figure 2B) uses a chelating pentadentate monoanionic nitrogen-donor ligand, including a carboxamido donor, highlighting that a ligand scaffold containing negatively charged groups can help stabilize the metal center at high oxidation states. In this report, oxygen evolution catalysis was carried out with a chemical oxidant (hydrogen peroxide or oxone) rather than electrocatalytically [22]. However, anionic donors are not strictly necessary, as evidenced by the report from Smith of a dicationic mononuclear manganese pyridinophane complex (Figure 2C) that is effective in electrocatalytic water oxidation (TON = 16–24) [23]. Scattered examples of other mononuclear manganese complexes that are competent precatalysts for electrocatalytic water oxidation, often supported by polydentate nitrogen donor ligands, have been reported in the last few years, although in some cases, the nature of the active catalyst in the system is not yet clear [24,25,26,27].

**Figure 3:**
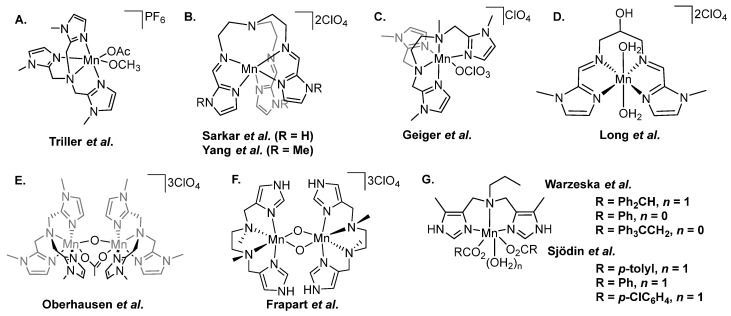
Selected examples of manganese complexes of polydentate nitrogen-donor ligands, including multiple imidazole donors [33,34,35,36,37,38,39,40,41]. All examples are crystallographically characterized except for (**C**) [37], whose structure is hypothesized by analogy to the analogous polypyridyl complex.

In synthetic biomimetic ligands, as observed in several of the water oxidation systems discussed above, pyridine donors often serve as “stand-ins” for imidazoles. Although certainly chemically similar, there are key differences between pyridine and imidazole donors that could impact the reactivity of the resulting complexes; importantly, imidazoles are stronger donors than pyridine, as indirectly illustrated by the aqueous pK_a_ values of their conjugate acids—5.23 for pyridinium and 6.95 for imidazolium (~6.0 for the side chain in histidine, specifically) [28]. In some recent examples, researchers have taken advantage of this difference by replacing pyridine donors with imidazoles, resulting in, for instance, enhanced water oxidation catalysis using a tetraimidazolyl–pyridine cobalt complex [29]. Earlier examples include the use of imidazole donors in scorpionate-type ligands in an effort to mimic zinc metalloenzymes such as carbonic anhydrase and the incorporation of imidazole donors into a phenol-bridged dinucleating ligand designed to mimic bimetallic metalloenzyme sites [30,31,32]. In the latter case, a mixed-valent Fe^II/III^ complex of the imidazole-appended ligand was found to be significantly easier to oxidize (by about 250 mV) than an analogous complex of a ligand bearing pyridine donors instead, bolstering the aforementioned point that imidazoles can be stronger electron donors [32]. Manganese complexes supported by polyimidazole-containing nitrogen-donor ligands have long been explored in the context of biomimetic redox processes, although they still remain somewhat less thoroughly investigated than related pyridine-containing scaffolds; selected examples are shown in Figure 3 [33,34,35,36,37,38,39,40,41].

In water oxidation catalysis and any catalysis requiring strongly oxidizing conditions, the stability of the catalyst under the reaction conditions becomes a major concern. Many common organic ligands are prone to oxidative degradation, and design rules have been proposed to improve ligand stability under oxidizing conditions [42,43]. However, it is noteworthy that some of the previously explored examples of mononuclear manganese complexes for water oxidation, such as those reported by Brudvig (Figure 2B) and Smith (Figure 2C), bear vulnerable C-H bonds (α to heteroaromatic rings) that are likely highly susceptible to oxidation.

**Scheme 1:**
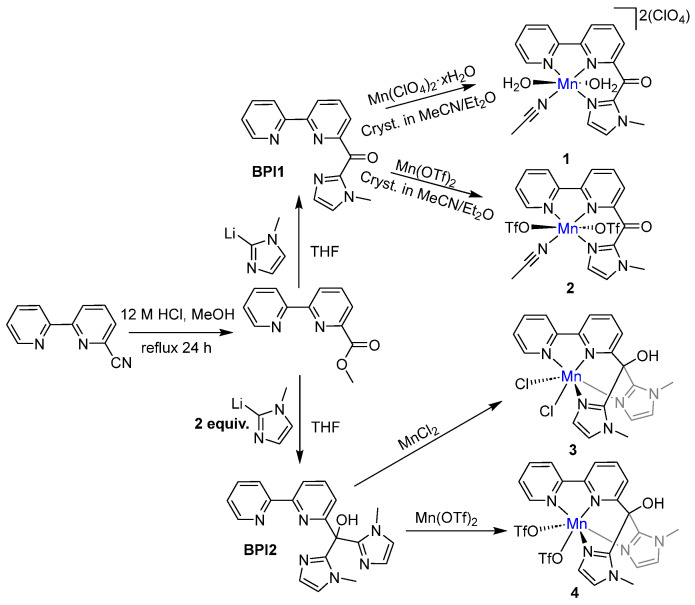
Synthetic routes to bpy-(imi)_n_ ligands BPI1 and BPI2, and complexes **1**–**4**.

Given the above discussion, we sought to develop and test (in catalytic oxidations) new polydentate N-donor ligands for manganese that include biomimetic imidazole donors, have the potential to be oxidatively stable due to a lack of vulnerable C-H bonds, and give rise to a manganese center with *cis*-disposed labile sites as observed in the biological systems mentioned above. Therefore, in this study, we designed and prepared a series of new bpy-(imidazole)_n_ (bpy = 2,2′-bipyridyl) ligands for n = 1 or 2, abbreviated BPI1 and BPI2, respectively (Scheme 1). Four complexes of these ligands with Mn^2+^ are structurally characterized using single-crystal X-ray diffraction. UV-vis absorption spectroscopy and cyclic voltammetry analyses of the complexes are consistent with ligand-centered reductions and metal-centered oxidations. Unfortunately, studies in solution (ESI-MS and potentiometric titration) suggest that the complexes have low stability in aqueous solution; the electrocatalytic water oxidation behavior in aqueous solution was tested, but the observed activity was found to result primarily from the deposition of a heterogeneous species on the electrode. We finish with a brief discussion of possible reasons for this instability and suggested future ligand modifications.

## 2. Results

### 2.1. Synthesis

Two ligand scaffolds were prepared based on a bipyridine backbone flanked by one (BPI1) or two (BPI2) additional N-methylimidazole donors. Scheme 1 shows the overall synthetic scheme used to access the ligands. Both ligands are derived by the addition of one or two equivalents of 1-methyl-2-lithioimidazole to 6-methoxycarbonyl-2,2′-bipyridine, following a procedure similar to that used previously to access related pyridine-containing ligands [29,44,45]. In practice, the reactions tend to give a mixture of the BPI1 and BPI2 products, but they can readily be separated chromatographically, and the ligands are obtained as purified products in yields of around 60% in both cases (see experimental section for detailed procedures). Metalation was carried out by combining various manganese(II) precursors (see Scheme 1) with a stoichiometric amount of ligand in refluxing ethanol, yielding a light yellow solution of complexes **1**–**4**; the compounds were isolated as microcrystalline powders following precipitation from acetonitrile/ether in yields ranging from 76% to 92%. Note that complex **1** crystallizes as a 2:1 mixture of mono(aqua) and bis(aqua) complexes (vide infra); for simplicity, only the bis(aqua) complex is shown in Scheme 1. The compounds had magnetic moments (measured using Evans method in d_3_-acetonitrile solution) of 5.9–6.1 µ_β_, close to the expected value for high-spin manganese(II) centers; as is common for such compounds, no discernible signals were observed in the ^1^H NMR spectra of **1**–**4** (d_3_-acetonitrile, 500 MHz, 298 K). Room temperature solution electron paramagnetic resonance (EPR) spectra showed the expected sharp 6-line hyperfine split pattern near g = 2.0 in each case (Figure S8).

### 2.2. X-ray Crystallography

**Figure 4:**
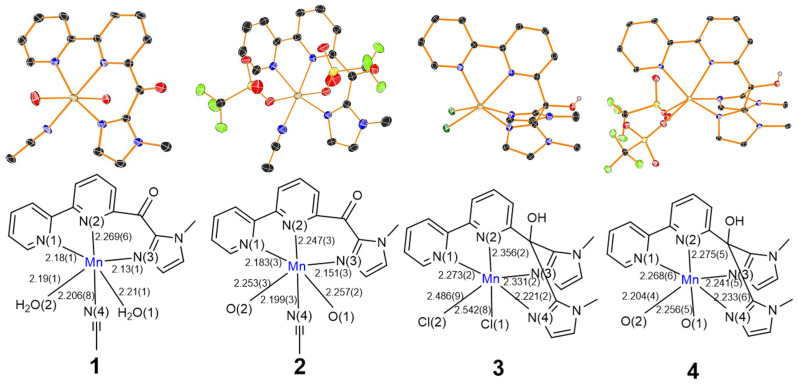
X-ray crystal structure of complexes **1**–**4**, with ellipsoids shown at the 50% probability level and hydrogen atoms on carbon, are omitted. Two perchlorate counteranions in **1** are omitted, and only one of three crystallographically inequivalent molecules is shown. To better visualize the geometric differences, the bottom stick diagrams show bond lengths in Å, with esd values in parentheses.

Mn^2+^ complexes **1**–**4** were characterized crystallographically. The growth of X-ray quality single crystals of complexes **1**–**4** was achieved using vapor diffusion of diethyl ether into acetonitrile or ethanol. The structures of all four complexes were confirmed by single-crystal X-ray diffraction and are shown in Figure 4. Refinement data are summarized in Table S1, with selected bond lengths and bond angles summarized in Table S2.

In all cases, the manganese metal center resides in the center of a distorted octahedral coordination geometry with one bpy–(imidazole)_n_ ligand and two or three solvent/counteranion molecules. In the BPI1 complexes **1** and **2**, the ligand binds as a tridentate, nearly planar meridional NNN pincer ligand. One coordinated acetonitrile ligand lies trans to the central pyridine ring in the ligand plane. The refined structure of complex **1** was found to contain three crystallographically inequivalent molecules; in one of the molecules, two water molecules bind in the remaining axial sites at the octahedral manganese center, while for the other two molecules, the coordination sphere is completed by one water molecule and one perchlorate. The bis(aqua)-ligated complex is shown in Figure 4; the other two molecules have similar metrical parameters and are shown in the Supplementary Information (Figure S1). For complex **2**, two triflate anions complete the coordination sphere in a mutually trans disposition. Complexes **1** and **2** have similar bond metrics with respect to the BPI1 ligand, such as the Mn–N1_N^N_, Mn–N2_N^N_, and Mn–N_imi_ bond distances (2.132(8)–2.265(8) Å) as well as the N-Mn-N bipyridine chelate angles, which span from 75.1(3)° to 76.3(1)°. The bond lengths to the central pyridine rings are slightly longer than those of the flanking pyridine and imidazole donors (see Figure 4) [29,46,47].

As seen in the structures of complexes **3** and **4**, the BPI2 ligand binds in a tetradentate fashion, occupying four sites in a distorted six-coordinate geometry. This ligand geometry leaves two labile, cis-disposed sites available for the coordination of solvents, counterions, or substrates, akin to the Mn^2+^-binding cupin protein shown in Figure 1A. It has been suggested that this type of chelate geometry may be particularly favorable for accelerating key steps in certain catalytic reactions, such as the O-O bond-forming step in water oxidation [6,7,8,9]. Both BPI2 complexes **3** and **4** exhibit slightly longer bond distances to the chelating ligand as compared with the BPI1 complexes, with Mn-N distances ranging from 2.221(2)–2.356(2) Å. Notably, the chloride complex **3** has two particularly long bond lengths, with the Mn-N bond to the central pyridine ring at 2.356(2) Å and the Mn-N bond to the imidazole ring trans to one of the chloride ligands at 2.331(2) Å, possibly due to the slightly stronger trans influence of the chloride ligands relative to the triflate ligands in **4**. The two imidazole chelating N-Mn-N angles, with a span of 78.93(8)–83.77(19)°, are similar to those in previously reported pyridine–tetraimidazole Co^2+^ complexes [29,48], suggesting that similar binding modes occur among the first-row transition metals in this general class of ligands.

In all cases, the constraints of the chelating ligand result in distortions from an ideal octahedral coordination geometry. This can be quantified using the angular distortion parameters Σ and Θ, which relate to the ligand–metal–ligand and torsional angles around the metal center, respectively; both values are zero for a perfect octahedron and higher values indicate more distortion [49]. While the BPI1 complexes are moderately distorted (Σ ~ 45–70° and Θ ~ 170–235°; see Table S3 for exact values), the complexes of the tetradentate BPI2 ligand (**3** and **4**) are severely distorted to the extent that they are no longer well-described as octahedral (Σ ~ 190° and Θ > 1100°). This is due to the constraints of the chelating BPI2 ligand; the ligand itself enforces a coordination environment that can roughly be described as trigonal monopyramidal, with the ancillary ligands (Cl^−^ or OTf^−^) occupying the remaining open pseudo-axial site(s).

### 2.3. Solution Speciation

The labile nature of metal–ligand bonds for high-spin manganese(II) complexes means that the nature of complexes in solution may differ from that in the solid state (as observed in the crystal structures), and multiple species may exist in equilibrium in solution. For example, as is generally observed in related complexes, the labile anions in complexes **1**, **2**, and **4** (perchlorate or triflate) are likely displaced in the presence of coordinating solvents such as acetonitrile and water; this is already observed in the crystal structure of complex **1** where a mixture of perchlorate and solvent coordination was observed. Furthermore, aquated Mn^2+^ tends to bind relatively weakly to most ligands relative to other divalent first-row transition metals, making the preparation of strongly bound complexes in water solution challenging [50]. Since we were ultimately interested in applying these complexes in catalytic reactions, we sought to gain some insight into the solution speciation of manganese complexes of BPI1 and BPI2 using high-resolution electrospray ionization mass spectrometry (ESI-MS) of solutions of these complexes in acetonitrile and mixed acetonitrile/water solutions.

**Figure 5:**
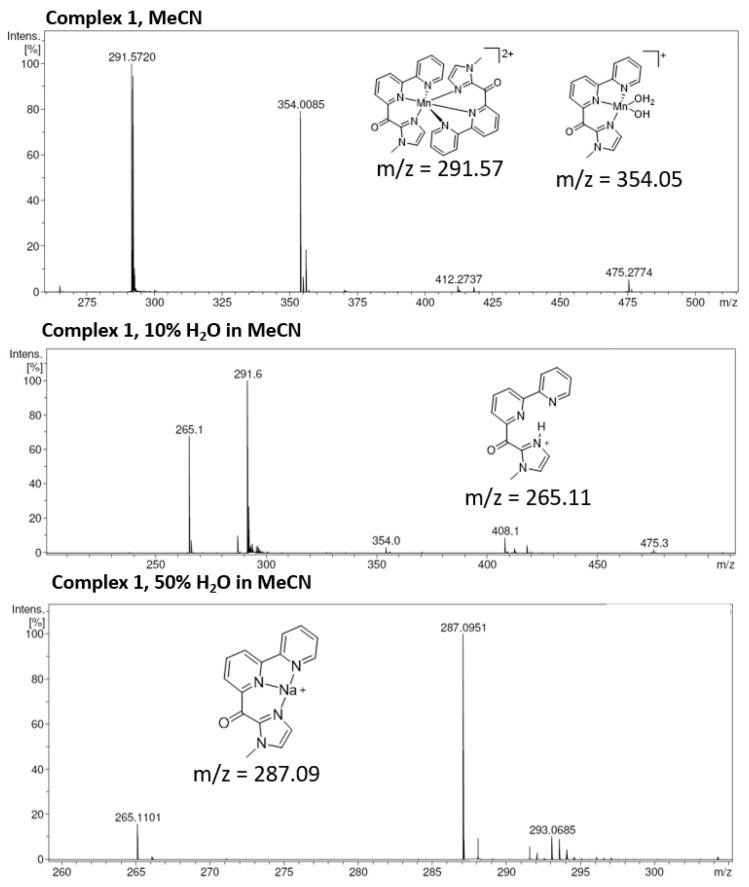
Mass spectrometry (ESI-MS) data for solutions of complex **1** (60 µM).

Complex **1** was studied as representative of the coordination behavior of BPI1 in solution. The complex was dissolved at a 60 µM concentration in acetonitrile, 10% (vol/vol) water in acetonitrile, or 50% water in acetonitrile. The resulting ESI-MS data are shown in Figure 5. Note that due to instrumental limitations, peaks below m/z = 200 amu can not be observed. In pure acetonitrile, two prominent mass peaks are observed. One at m/z = 354.05 amu can be attributed to a (BPI1)Mn(OH_2_)(OH)^+^ cation. Unexpectedly, a strong signal was also observed at m/z = 291.57 amu that could be attributed to a bisligated Mn^2+^ ion (BPI1)_2_Mn^2+^ (see Figure S25 for expanded view). This observation suggests that ligand redistribution occurs in solution and that a significant population of such a bis-ligated species may be present when the complex is dissolved in acetonitrile, despite the fact that both complex **1** and complex **2** crystallized in the solid state with a 1:1 ligand:metal ratio. In pure acetonitrile, only a negligible amount of free ligand (m/z = 265.11) was observed.

When 10% water was added to the solution, the bis-ligated (BPI1)_2_Mn^2+^ cation was still observed, along with the free ligand at m/z = 265.11. This suggests that even in only partially aqueous solution, the ligand can be displaced from manganese by H_2_O. When the water content is increased to 50%, only the free ligand (BPIH^+^ or BPINa^+^) is substantially observed; no strong signals corresponding to the Mn^2+^-coordinated ligand could be identified. These observations are consistent with weak binding of BPI1 to Mn^2+^ in the presence of water.

**Figure 6:**
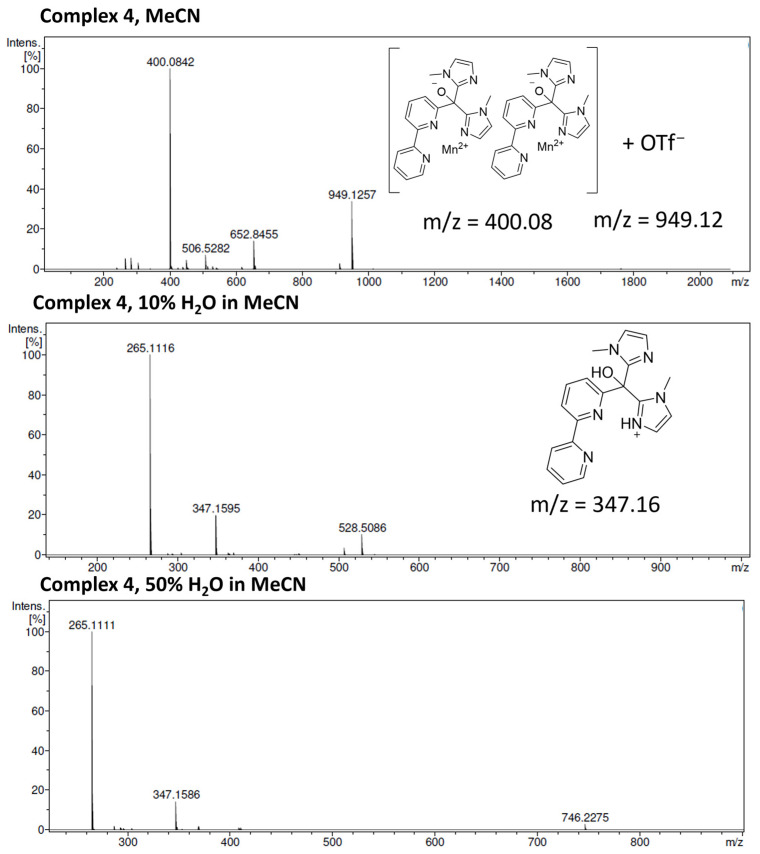
Mass spectrometry (ESI-MS) data for solutions of complex **4** (30 µM).

The tetradentate nature of BPI2 might be expected to disfavor the formation of bis(ligated) manganese complexes and perhaps to increase the stability of the metal–ligand complexes in the presence of water. Complex **4** was studied by ESI-MS to probe the speciation of BPI2 with Mn^2+^ in acetonitrile, 10% water in acetonitrile, and 50% water in acetonitrile solutions (30 µM concentration). Interestingly, the results (Figure 6) suggest that there may be a significant formation of clusters of at least two BPI2Mn^2+^ units. In pure acetonitrile, the dominant peak at m/z = 400.0842 amu corresponds well to the expected mass and isotope pattern of a species consisting of two Mn^2+^ ions and two BPI2 ligands, presumably deprotonated. While we cannot determine from these data what the specific structure of this cluster would be, we hypothesize that it could involve coordination of the deprotonated alkoxides to Mn^2+^. A second strong signal at m/z = 949.1257 amu is consistent with the same species combined with a triflate anion. Other lower-intensity signals in the mass spectrogram could not be definitively identified. When 10% water is incorporated into the solution, the strongest peaks observed now correspond to free ligands, including m/z = 347.1595 amu for BPI2H^+^ and m/z = 265.1116 amu, which corresponds to the mass of BPI1H^+^ (vide supra), likely representing a facile fragmentation pathway of the free ligand in the presence of water; other minor peaks could not be definitively identified. Similarly, at 50% water content, only the free ligand peaks are observed.

**Figure 7:**
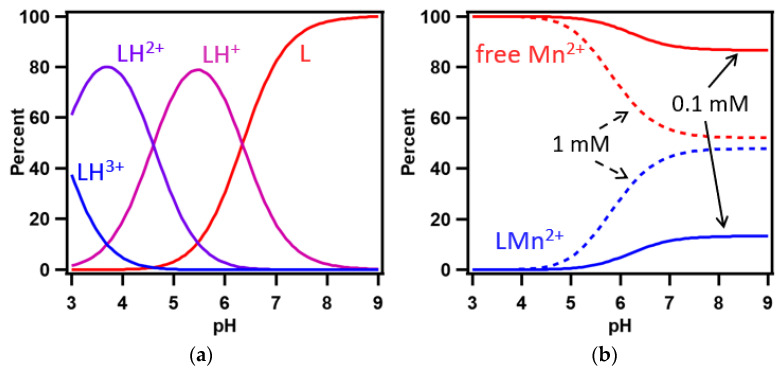
Speciation curves for BPI2 (L = BPI2) as a function of pH in aqueous solution based on the stability constants given in Table 1. (**a**) Speciation curves for the ligand showing the percentage of different protonation states; simulated for a total ligand concentration of 0.1 mM. (**b**) Speciation curves for Mn^2+^ in the presence of equimolar BPI2, simulated at two different concentrations (1 mM = dashed lines; 0.1 mM = solid lines).

These ESI-MS data suggest that the association of BPI2 with Mn^2+^ is weak and readily displaced in the presence of water. To further quantify this, the association of BPI2 with Mn^2+^ in water was also probed by determining the ligand–metal association constant in aqueous solution using potentiometric titration. The ligand protonation constants and metal–ligand association constant (K_Mn_) are given in Table 1, and the speciation curves for Mn^2+^ are shown in Figure 7; raw data and speciation curves for the ligand are shown in Figures S14 and S15. The low association constant of log(K_Mn_) = 3.25 suggests that, especially at relatively dilute concentrations, the majority of the manganese ions in solution will not be coordinated by the ligand. This is demonstrated by the simulated Mn^2+^ speciation curves (Figure 7) at 1 µM and 0.1 µM concentrations, where only approximately 50% or 10% of the Mn^2+^ present, respectively, is bound to BPI2 at neutral pH. We note that this analysis and fitting of these potentiometric titration data are somewhat oversimplified since they do not account for the more complex speciation of **4** (e.g., the formation of clusters) observed by ESI-MS but should still provide a reasonable estimate of the speciation of free vs. coordinated Mn^2+^ in solution.

### 2.4. UV-Vis Absorption Spectroscopy

**Figure 8:**
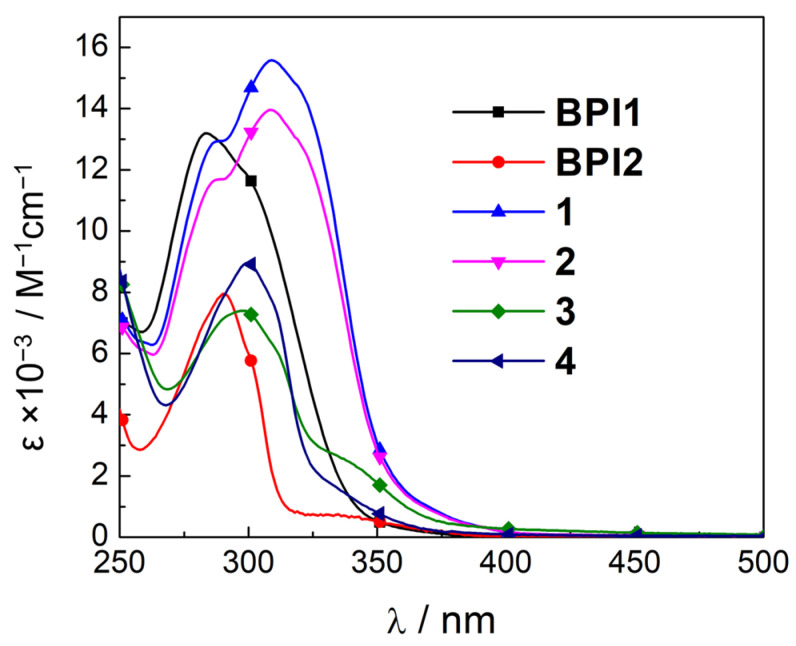
Overlaid electronic absorption spectra of bpy–(imidazole)_n_ ligands and complexes **1**–**4** in acetonitrile solution. Spectra were recorded in acetonitrile solution at room temperature. Data were collected in intervals of 1 nm, and symbols were included on each plot to help distinguish the overlaid spectra.

Complexes **1**–**4** appear nearly colorless in solution. UV-vis spectra for the ligands (BPI1, BPI2) and their complexes are shown in Figure 8, with a summary of data in Table 2. The ligands and their complexes show similar intense transitions near 300 nm, indicating that these major absorption features are primarily ligand-centered, localized π→π* transitions, although there may be some metal–to–ligand charge-transfer (MLCT) contributions, especially in the higher-energy region (vide infra) [51]. The free BPI1 ligand shows a dominant absorbance peak at 283 nm accompanied by a small shoulder at 299 nm. The BPI1 complexes (**1**,**2**) have three clear features in this region and display essentially identical spectra, consistent with the fact that the labile triflate and solvent ligands present in the isolated crystal structure are likely displaced in solution, leading to identical metal-containing fragments that differ only in their outer-sphere counterions (vide supra). The spectra show a strong absorbance feature redshifted relative to the free ligand peak (283 nm for BPI1 → 309 nm for **1** and **2**), also accompanied by a small shoulder on the low-energy side (320 nm). An additional peak at 287 nm is also apparent.

The series of BPI2 ligand and complexes (**3** and **4**) have a slightly weaker molar absorptivity compared to BPI1, and the complexes similarly manifest a red shift of the main absorption peaks upon binding (λ_max_ = 291 nm (BPI2) → 298 nm (**3**)/300 nm (**4**)). However, the shift is smaller than for **1** and **2,** and the resulting peaks are at shorter wavelengths; this may be consistent with a somewhat weaker interaction with the bipyridine moiety in these ligands, as suggested by the longer bond lengths observed crystallographically. A notable low-energy absorption feature at about 340 nm can be observed for complexes **3** and **4**, which is not as apparent in **1** or **2**, although it may be obscured in those cases by the major peak being shifted to longer wavelengths.

Given that our ESI-MS results suggest that multiple species are present in solutions of **1** and **4**, the UV-Vis spectra presumably represent a combination of signals from several such species. However, our computational results (see below) suggest that, with the exception of the chloride ligands in **3**, the auxiliary ligands participate negligibly in the transitions associated with the UV-Vis in the measured region, and many of the species proposed to be present are expected to show very similar spectra.

**Figure 9:**
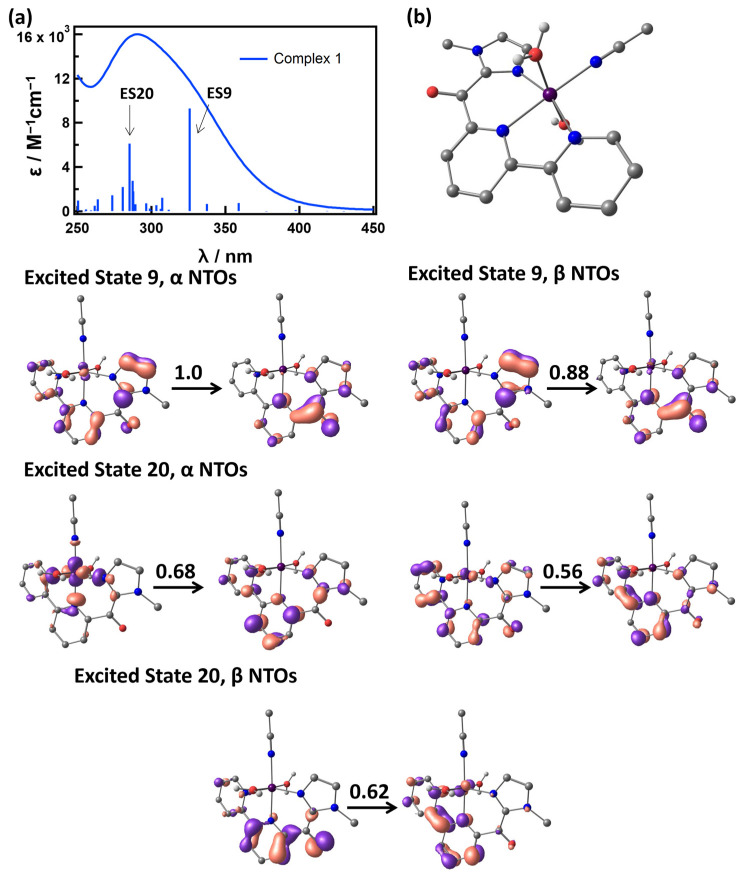
Calculated spectrum (**a**) and selected natural transition orbitals (NTOs) for the dication of complex **1** with two water ligands and one acetonitrile ligand. The optimized structure of the dication is shown in panel (**b**). Transitions between the dominant NTOs for the selected excited states are shown; numbers above the arrows indicate the occupancy of the corresponding NTOs, which is indicative of their relative contribution to the transition. Orbital contours are shown at an isovalue of 0.06.

Calculated spectra give some additional insight into the origin of the UV-Vis features. Time-dependent density function theory (TD-DFT) calculations were carried out on DFT-optimized structures for ligands BPI1 and BPI2, the cation of complex **1**, complex **3**, and a (BPI2)Mn(H_2_O)_2_^2+^ cation representing a possible solvated solution structure of **4**, and natural transition orbitals (NTOs) were generated and visualized [52]. Calculated spectra for the ligands are given in the Supporting Information (Figures S12 and S13). The calculated spectra qualitatively reproduce the experimental results, although the energies of the peaks are slightly overestimated (e.g., λ_max_ for BPI2 occurs at 269 nm). However, comparative features of these spectra, such as the slight shift to lower energies for the complexes relative to their ligands and the lower energy peaks of complex **1** relative to complex **3**, are well preserved. These calculations confirm that the observed features primarily involve the ligand π system, with some contribution from NTOs with partial MLCT character and the chloride ligands in complex **3**. Complex **1** shows two dominant features (which overlap significantly when Gaussian broadening is applied); the lower energy feature (excited state (ES) 9) is almost entirely ligand-based, delocalized over the π system of all three rings, and with significant n → π* character on the carbonyl group (Figure 9). The higher energy feature contains overlapping contributions from multiple excited states, dominated by ES20; while this transition also has considerable ligand π → π* and n → π* character, there is a large contribution from an NTO with MLCT character involving a d_x2-y2_ type orbital on Mn^2+^.

**Figure 10:**
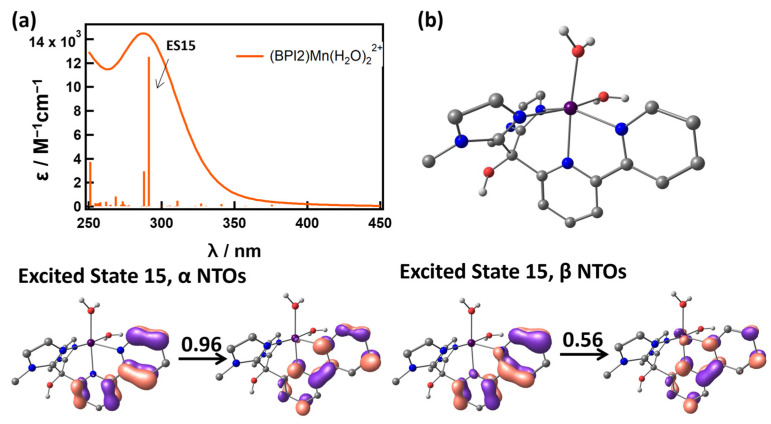
Calculated spectrum (**a**) and selected natural transition orbitals (NTOs) for the (BPI2)Mn(H_2_O)_2_^2+^ dication. The optimized structure of the dication is shown in panel (**b**). Transitions between the dominant NTOs for the selected excited state are shown; numbers above the arrows indicate the occupancy of the corresponding NTOs, which is indicative of their relative contribution to the transition. Orbital contours are shown at an isovalue of 0.06.

**Figure 11:**
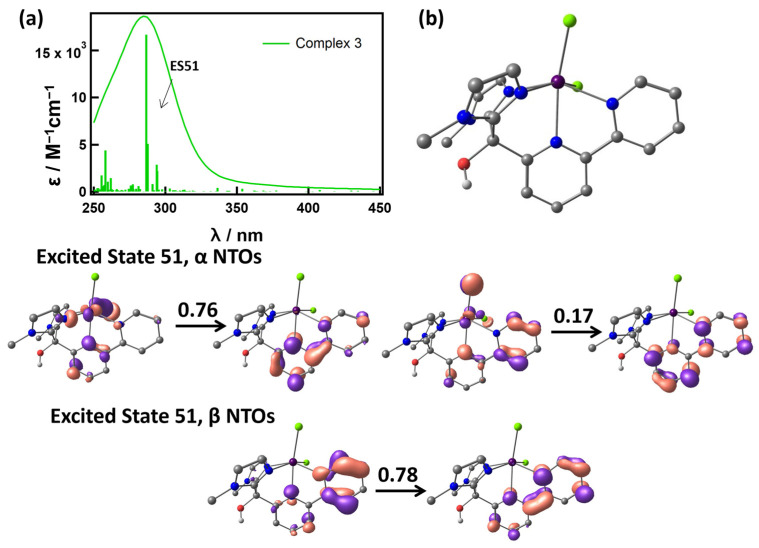
Calculated spectrum (**a**) and selected natural transition orbitals (NTOs) for complex **3**. The optimized structure of the complex is shown in panel (**b**). Transitions between the dominant NTOs for the selected excited state are shown; numbers above the arrows indicate the occupancy of the corresponding NTOs, which is indicative of their relative contribution to the transition. Orbital contours are shown at an isovalue of 0.06.

The spectra of the (BPI2)Mn(H_2_O)_2_^2+^ complex are dominated by one feature in the region of interest, corresponding primarily to ES15 (Figure 10). The NTOs making this transition are entirely π → π* in character and localized on the bipyridine moiety, with negligible contribution from Mn^2+^ or the water ligands. In contrast, while the spectrum of complex **3** is similarly dominated by one peak, corresponding primarily to ES51, the transition has significant MLCT/LLCT character involving chloride lone pairs and Mn^2+^ d_x2−2_ and d_xz_-type orbitals (Figure 11). As in (BPI2)Mn(H_2_O)_2_^2+^, the ligand π system contributions to the spectrum of complex **3** in the measured region are confined to the bipyridine moiety, with negligible contribution from the imidazole rings.

The UV-Vis spectra of complexes **1** and **4** (0.1 mM) were also measured in acetonitrile/water mixtures containing up to 50% water. In both cases, the resulting spectra (Figures S20 and S21) show a shift to higher energies as water is added, suggesting the presence of increasing amounts of free ligand as the water content increases. This is consistent with the ligand dechelation observed in ESI-MS data.

### 2.5. Electrochemistry

**Figure 12:**
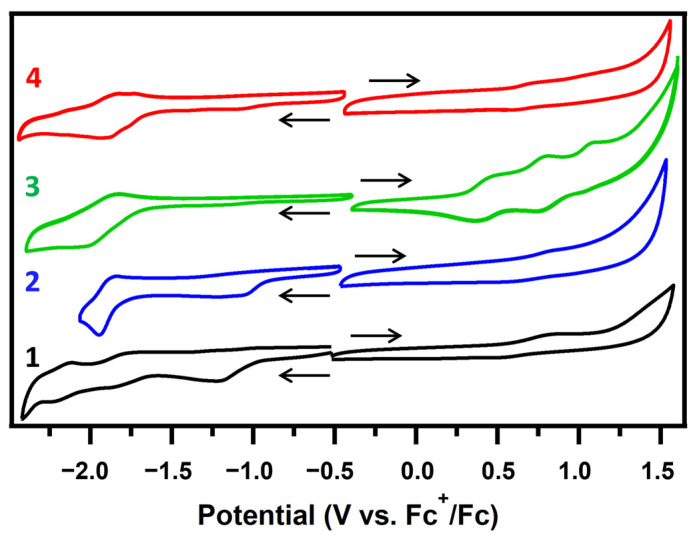
Cyclic voltammograms of complexes **1**–**4**, vertically offset for clarity. CVs were recorded in acetonitrile with 0.1 M NBu_4_PF_6_ supporting electrolyte, using a glassy carbon working electrode and a scan rate of 100 mV/s. The arrows indicate the scan direction. The potential was internally referenced to Fc/Fc^+^ after the measurement.

**Figure 13:**
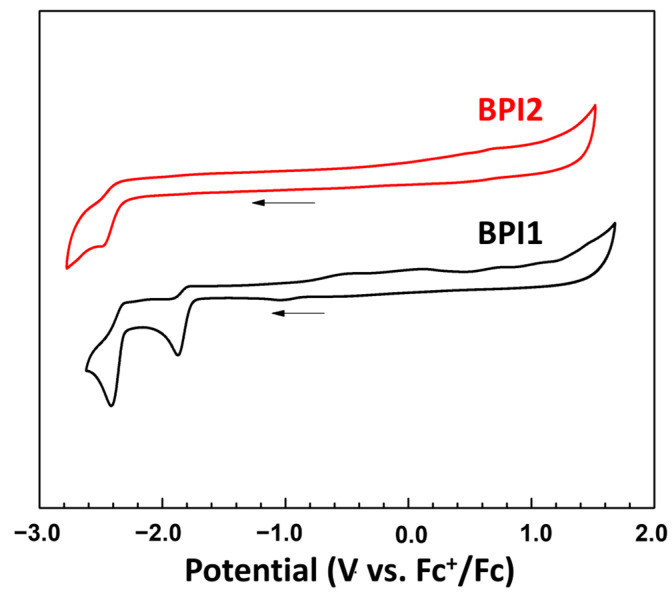
Cyclic voltammograms of ligands BPI1 and BPI2, vertically offset for clarity. CVs were recorded in anhydrous acetonitrile with 0.1 M NBu_4_PF_6_ supporting electrolyte, using a glassy carbon working electrode and a scan rate of 100 mV/s. The arrows indicate the scan direction. The potential was internally referenced to Fc/Fc^+^ after the measurement.

The redox properties of all complexes were evaluated by cyclic voltammetry (CV) in anhydrous acetonitrile with a 0.1 M NBu_4_PF_6_ electrolyte. CV traces are shown in Figure 12. Cyclic voltammograms of the free ligands have also been measured for comparison (Figure 13). For CVs taken in non-aqueous solution, all potentials are internally referenced to the ferrocene/ferrocenium (Fc/Fc^+^) couple.

Both ligands show irreversible reductions at strongly negative potentials (Figure 13). While both BPI1 and BPI2 have an irreversible reduction peaking at −2.5 V, only BPI1 has an additional reduction at −1.8 V. On the basis of this and comparison to bipyridine reduction potentials reported in the literature [53,54], the first reduction on BPI1 (−1.8 V) is attributed to reduction localized primarily on the ketone moiety, while the second reduction of BPI1 and only reduction of BPI2 at −2.5 V is attributed to reduction of the bipyridine moiety to give a bipyridine radical anion. Neither ligand shows any discernible oxidation features up to 1.5 V vs. Fc/Fc^+^.

The reductive features for complexes **1**–**4** are qualitatively similar to those observed for their ligands but shifted anodically as expected following metal coordination, suggesting that these reductions are also ligand-centered. The BPI1 complexes **1** and **2** have an irreversible reduction feature at E_pc_ = −1.19 (**1**) or −1.07 (**2**), which most likely corresponds to the ketone-centered reduction observed for the ligand, given that no analogous feature is observed in the BPI2 complexes **3** and **4**. Compound **2** has a second reduction at E_1/2_ = −1.90 V, which appears reversible, suggesting that the bipyridine-centered reduction observed in the ligand becomes more reversible upon metal coordination, presumably due to stabilization of the resulting bipyridine radical anion; the analogous feature is less well-defined in the sample of compound **1**.

The BPI2 complexes **3** and **4** both show one dominant redox feature near −2.0 V, again attributed to the ligand-centered reduction of the bipyridine moiety and anodically shifted from the free ligand reduction as expected. The smaller anodic shift of this feature for the BPI2 complexes relative to the BPI1 complexes may again be related to the weaker interaction of the Mn^2+^ centers with the bipyridine ligand, as suggested by the crystal structures and UV-vis absorption (see above).

On the oxidative side, the behavior of the is dependent on the co-ligands/counterions on the metal center. Given this and the lack of oxidative features in the free ligand CVs, we attribute all oxidative features in the complexes to metal-centered oxidations. For the BPI1 complexes, significant oxidative waves are not observed; however, a small oxidative feature which could be related to the presence of trace water is present at approximately 0.8 V; note that although anhydrous solvent and electrolyte were used for the electrochemistry, complex **1** was isolated with coordinated water ligands. For the BPI2 complexes, complex **3** (chloride co-ligands) shows multiple redox features, suggesting that multielectron oxidation may be accessible on this scaffold, although we have not characterized the oxidation products. However, no well-defined oxidation features are observed in the triflate complex **4**, suggesting that the presence of the more strongly-donating chloride ligands in **3** is important in enabling oxidation under these conditions. It should be noted that although complexes **2** and **4** crystallize with coordinated triflate ions, it is likely that these are displaced by acetonitrile in solution, as discussed above. Displacement of the triflate ligands to give rise to a dicationic species would be consistent with the lack of accessible oxidation features. Large shifts in oxidation potentials (Δ = 0.5 V) upon exchange of chloride ligands for acetonitrile have been observed in other studies of putative first-row transition-metal water oxidation catalysts supported by neutral polydentate nitrogen-donor ligands, consistent with our observations of a dramatic effect of co-ligands on the oxidation behavior [55,56].

The orbital nature of the reduction and oxidation processes (ligand- vs. metal-centered) was also supported by our DFT calculations on the corresponding species (complex **1** with water ligands, complex **3**, and BPI2Mn(MeCN)_2_^2+^, which was chosen as representative of the solution speciation of complex **4** as noted above). In all cases (Figures S9–S11), the lowest unoccupied molecular orbitals (LUMOs) are ligand-centered, and the highest occupied molecular orbitals (HOMOs) are metal-centered. The HOMO of complex **3** also includes significant contribution from the chloride ligands, whereas the ancillary water and acetonitrile ligands in the other complexes have only minor contributions, consistent with the different oxidation behavior observed for this complex; the HOMO of complex **3** also lies at significant higher energy than those of the other complexes.

Given that these complexes show evidence for accessible metal-centered oxidations, albeit dependent on the nature of the coligands on Mn^2+^, we next sought to probe whether they were competent in electrochemical water oxidation catalysis.

### 2.6. Electrochemistry in the Presence of Water

**Figure 14:**
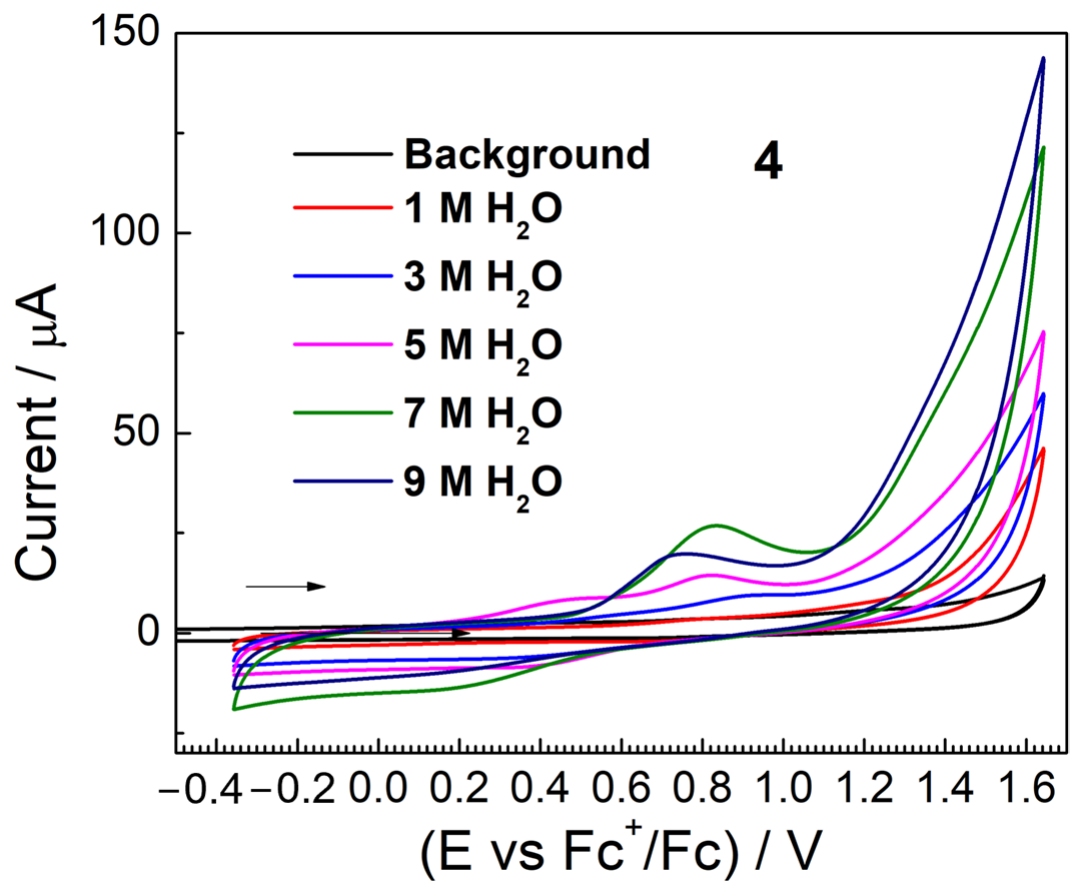
Overlaid cyclic voltammograms of complex **4** (0.24 mM) with increasing amounts of H_2_O in acetonitrile with 0.1 M NBu_4_PF_6_ supporting electrolyte, using a glassy carbon working electrode and a scan rate of 100 mV/s. The arrow indicates the scan direction. The background scan contains no complex and no water.

Since our speciation studies showed that BPI1 complexes likely substantially redistribute in solution to give bis-ligated Mn^2+^ species, we have focused further studies primarily on the BPI2 complexes; for this ligand, our studies (discussed above) suggested a 1:1 L:Mn^2+^ complexation is maintained even if the speciation in solution may still be complex and involve the formation of multinuclear clusters. BPI2 complexes **3** and **4** were subjected to further study to probe how their electrochemical behavior was influenced by the presence of small amounts of water. First, measurements were carried out in acetonitrile (0.1 M NBu_4_PF_6_) with increasing amounts of added water, using a glassy carbon (GC) working electrode; the resulting overlaid cyclic voltammograms for complex **4** are shown in Figure 14. Both complexes show significant changes to their electrochemical behavior upon the introduction of water. For complex **4** (triflate co-ligands), the addition of water causes a new irreversible oxidation to appear at about 0.8 V, suggesting that coordination of water changes the oxidation behavior of the complex, facilitating an Mn(II)/Mn(III) couple that was not accessible for the complex in the absence of water. Bound water ligands commonly act to facilitate the oxidation of metal centers due to their ability to undergo proton-coupled electron transfer (PCET), allowing “redox leveling,” and this irreversible pre-wave could correspond to an Mn(II)-OH_2_ → Mn(III)-OH + H^+^ + e^−^ process [55]. We further observe a significant increase in current at higher potentials (onset ~1.1 V), which grows with further addition of water. This could be associated with a catalytic process involving the oxidation of water; this possibility is explored further below.

For complex **3** (chloride co-ligands), the oxidative waves that were originally observed in dry acetonitrile (Figure 12) disappear upon the addition of water and are replaced with a new quasireversible oxidation feature at E_1/2_ = 0.75 V (with 1 M H_2_O), which further shifts cathodically and broadens as the water concentration is increased (Figure S16). At higher potentials, a strong increase in current is again observed at a 1 M H_2_O concentration, but only a small further increase is observed as the water concentration is increased. Since complexes **3** and **4** are supported by the same chelating BPI2 ligand, the differences in their behavior in these experiments are presumably related to the presence of chloride ligands in **3**. Some observed features could be related to chloride oxidation, as previously observed under similar conditions in the study of related systems [55].

### 2.7. Attempted Water Oxidation Electrocatalysis in Aqueous Buffer Solutions

**Figure 15:**
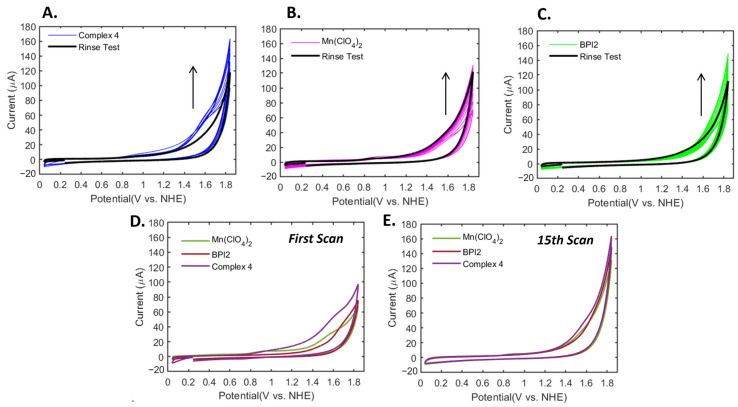
Electrochemistry of complex **4**, Mn(ClO_4_)_2_, and BPI2 in water. All were at a concentration of 0.24 mM in 0.1 M phosphate buffer (pH = 7), using a 3 mm glassy carbon working electrode, platinum wire counter electrode, and an Ag^+^/AgCl reference electrode. Arrows show the direction of change in current upon subsequent scans. (**A**–**C**) Fifteen scans of **4**, Mn(ClO_4_)_2_, and BPI2, respectively, overlaid with the first scan of a lightly rinsed electrode placed in a fresh buffer solution. (**D**) Overlay of the first scan of each analyte taken on a freshly polished electrode. (**E**) Overlay of the 15th subsequent scan for each analyte.

We further tested the electrochemical behavior of **4** in aqueous phosphate buffer at pH 7 (note that aqueous CVs and potentials are shown referenced to NHE). We expect only a fraction of the Mn^2+^ ions to be coordinated (~20% at a 0.24 mM concentration at pH 7, based on simulations using our determined K_Mn_), which is supported by the UV-vis spectra of complex **4** in the aqueous buffer solution, which is only slightly shifted from the free ligand spectrum (Figure S23). Nevertheless, we were still interested in determining if any catalytic water oxidation activity could be attributed to this complex in solution. Unfortunately, we could find no unambiguous evidence of water oxidation catalysis by complex **4** or other BPI2-ligated manganese species. For a 0.24 mM solution of **4**, an increase in oxidative current is observed with an initial onset near 0.9 V and a rapid rise beginning near 1.3 V vs. NHE, with further current increases observed upon subsequent scans, suggestive of the deposition of an electrochemically active species on the surface of the electrode (Figure 15A). After 15 scans, a rinse test was performed (the electrode was lightly rinsed with deionized water and then placed into a fresh buffer solution); significant oxidative current was still observed (about 75% of the current observed for the previous scan; Figure 15A). This suggests that the majority of the observed current can be attributed to an electrode-deposited species. We note that controlled potential electrolysis studies confirm the formation of O_2_ from the electrolysis of solutions of complex **4** (see Supplementary Materials, Figures S22 and S24).

A simple manganese salt, manganese(II) perchlorate, showed very similar behavior under the same conditions (Figure 15B). In the case of Mn(ClO_4_)_2_, the current after the rinse test remained almost identical rather than decreasing somewhat, as was observed for complex **4**. The solution of complex **4** does show enhanced current relative to Mn(ClO_4_)_2_ on the first oxidative scan, especially at moderate potentials (e.g., between 1.2–1.7 V, see Figure 15D); however, after 15 scans, their behavior is nearly identical (Figure 15E). This suggests that even if some of the initially observed electrochemical activity can be attributed to a BPI2-Mn^2+^ complex, it rapidly degrades. Notably, we also found that a solution of the free ligand BPI2 shows some oxidative current in this region and forms an electrode-deposited species upon subsequent scans (Figure 15C). Therefore, the electrochemical behavior of a solution complex **4** in an aqueous solution can likely largely be explained as a combination of the contributions from ligand BPI2 and free Mn^2+^ rather than a ligated manganese complex, consistent with our conclusions above that the majority of Mn^2+^ would not be bound by BPI2 under these conditions. Studies at different pH values led to similar conclusions, generally showing evidence for even more substantial electrode deposition (Figure S18); additionally, studies of Mn^2+^ with varying amounts of added BPI2 ligand provided no evidence for a more stable Mn^2+^ complex in the presence of excess ligand (Figure S19).

## 3. Discussion

In this study, we describe the synthesis of two novel polydentate imidazole-containing ligands and characterize a series of bpy–(imidazole)_n_ manganese(II) complexes prepared using a general synthetic strategy. These ligands may find broader utility in complexes of other first-row transition metals due to their biomimetic nature and the ability of the tetradentate ligand, BPI2, to engender a rigid coordination environment with two *cis*-disposed vacant coordination sites. The geometries of the manganese complexes are supported by crystallographic characterization. Cyclic voltammetry of the complexes is consistent with ligand-centered reductions and metal-centered oxidations (in the case of a chloride-ligated complex); this suggests the possibility for these ligands to function as redox-active moieties in possible future applications. While the addition of small amounts of water to the complexes in acetonitrile promoted oxidation, catalytic water oxidation in aqueous solution is precluded by the low stability of these complexes in water. 

Although we have not yet attempted to study in detail the speciation and decomposition of the complexes under catalytic conditions at various pHs, we can hypothesize some reasons for their apparently low stability. It is well known that the binding affinity of chelating ligands to manganese(II) in aqueous solution tends to be relatively low (compared with other divalent first-row transition metals) [50,57]. The stability of this type of compound tends to be particularly low at non-neutral pH; under acidic conditions, it is possible that competitive protonation of the accessible basic sites on the ligand) results in the dechelation of the ligand. Under basic conditions, binding of hydroxide to one or both of the labile metal sites of **4** could weaken the ligand-metal association further; due to the relatively low steric bulk of the ligands, the formation of oxide-bridged species (LMn-O-MnL) is a plausible pathway en route to decomposition to metal oxide/hydroxide products.

The non-ideal binding geometry of the BPI2 ligand may also contribute to its lower stability. As noted above, the crystallographically characterized complexes of this ligand (**3** and **4**) showed a significantly distorted non-octahedral coordination environment. This can be attributed in part to the shape, size, and angles of the five-membered imidazole rings themselves; notably, a closely related previously reported tetrapyridine ligand shows a much less distorted coordination geometry (see Table S3; the stability of this complex was not discussed) [58].

Future ligand modifications for improved stability should likely include stronger donors, more favorable geometry, and possibly a higher denticity ligand, ideally without sacrificing the availability of two possible substrate coordination sites on the resulting complex: heptadentate and octadentate coordination at manganese(II) is possible and known on related systems, relying on appropriate design of the ligand geometry [59,60]. While more basic donors could be vulnerable to protonation leading to dechelation in an aqueous solution, careful ligand design could situate these donors in a sterically protected site where protonation would be kinetically disfavored. Greater steric protection around the metal site, for example, through the incorporation of bulkier substituents on the imidazoles, could also protect against multimetallic degradation pathways such as those that involve the initial formation of Mn-O-Mn linkages.

The low stability of complexes **1**–**4** in aqueous water oxidation electrolysis does not preclude their potential utility in other oxidative transformations, such as the oxidation of organic substrates for synthetic applications. In future studies, we plan to continue our exploration of polyimidazole manganese complexes and expand their utility in oxidative catalysis through continued tuning of the ligand environment.

## 4. Materials and Methods

### 4.1. Materials

Reactions were carried out in a nitrogen atmosphere using standard Schlenk techniques, while work-ups and crystallizations were carried out under ambient air unless otherwise stated. Solvents, starting materials, and reagents were of commercial origin and used without further purification unless stated otherwise below. Acetonitrile for UV-vis spectroscopy and electrochemical measurements was dried using the method of Grubbs [61], passing through dual alumina columns on a commercial solvent purification system (SPS). The acetonitrile was further dried by storage over 4 Å molecular sieves. Tetrabutylammonium hexafluorophosphate (TBAPF_6_) was recrystallized from hot ethanol, and ferrocene was sublimed at ambient pressure before use in electrochemical experiments. CDCl_3_ for NMR spectroscopy was stored over molecular sieves to remove acidic impurities and moisture. EPR spectra were recorded on a Bruker EMX-10 EPR (Bruker, Mannheim, Germany) at room temperature in a solution of EtOH/MeOH (4:1). The preparation of the ligands bpy–(imidazole)_1_ and bpy–(imidazole)_2_ was adapted from the synthesis of a related pyridine-containing ligand reported by Macmillion et al. [29].

### 4.2. Physical Methods

NMR spectra were recorded at room temperature on a 500 MHz Bruker AVANCE III and referenced internally using the residual solvent proton and carbon peaks. EPR spectra were recorded on a Bruker EMX-10 EPR at room temperature in a solution of EtOH/MeOH (4:1). UV–vis absorption spectra were recorded in acetonitrile solutions in screw-capped 1 cm quartz cuvettes using a Perkin Elmer Lambda 900 spectrometer (PerkinElmer, Inc., Waltham, MA, USA). Cyclic voltammetry (CV) measurements were performed with a CH Instruments potentiostat. Samples were dissolved in acetonitrile with 0.1 M TBAPF_6_ as a supporting electrolyte or in phosphate buffer for aqueous experiments. A 3 mm diameter glassy carbon working electrode and a platinum wire counter electrode were used. For non-aqueous experiments, a silver wire pseudoreference electrode was used, and the potential was internally referenced to Fc/Fc^+^. For aqueous experiments, an aqueous silver/silver chloride reference electrode from CH Instruments was used. O_2_ formation was analyzed using a Vernier Optical Dissolved Oxygen probe. The bulk purity for metal complexes and ligands is established using elemental analysis performed in a UNICUBE Elemental analyzer.

#### 4.2.1. 6-Cyano-2,2′-bipyridine

The title compound was prepared using a modified version of a literature procedure [62]. Benzoyl chloride (847 mg, 6.00 mmol) was added as a solution in dichloromethane (10 mL) dropwise over 2 min with stirring to a mixture of 2,2′-bipyridine-N-oxide (533 mg, 3.10 mmol) and KCN (700 mg, 10.7 mmol) in 11 mL H_2_O, and the mixture was stirred at room temperature overnight. Reaction completion was confirmed using TLC. The resulting dark red organic layer obtained after biphasic extraction from dichloromethane/water was dehydrated using MgSO_4_ and concentrated to dryness, and the solid was recrystallized from dichloromethane and pentane to attain the purified product. Yield: 470 mg (84%). ^1^H NMR (500 MHz, CDCl_3_) δ 8.70–8.65 (m, 2H, ArH), 8.46 (d, *J* = 8.0 Hz, 1H, ArH), 7.97–7.92 (m, 1H, ArH), 7.86 (td, *J* = 7.8, 1.8 Hz, 1H, ArH), 7.69 (dd, *J* = 7.6, 1.0 Hz, 1H, ArH), 7.39–7.35 (m, 1H, ArH). The spectroscopic properties of the compound were consistent with those reported in the literature [62].

#### 4.2.2. 6-Methoxycarbonyl-2,2′-bipyridine

The title compound was prepared using a modified version of a literature procedure [63]. Concentrated HCl (20 mL, 12 M) was added dropwise to the solution of 6-cyano-2,2′-bipyridine (400 mg, 2.20 mmol) in methanol (30 mL) and the reaction mixture was refluxed for 24 h under N_2_ atmosphere. The reaction completion was confirmed using TLC. After the reaction, methanol was removed via rotary evaporation, and the mixture was cooled to 0 °C. Saturated Na_2_CO_3_ aqueous solution was added to make the solution neutral (pH = 7). The resulting light yellow organic layer obtained after biphasic extraction from dichloromethane/water was dehydrated using MgSO_4_ and concentrated to dryness, and the solid was recrystallized from ethyl acetate and pentane to attain the purified product. Yield: 350 mg (75%). ^1^H NMR (500 MHz, CDCl_3_) δ 8.69 (d, *J* = 4.1 Hz, 1H, ArH), 8.61 (dd, *J* = 7.9, 0.8 Hz, 1H, ArH), 8.54 (d, *J* = 8.0 Hz, 1H, ArH), 8.14 (dd, *J* = 7.7, 0.8 Hz, 1H, ArH), 7.97 (t, *J* = 7.8 Hz, 1H, ArH), 7.85 (td, *J* = 7.8, 1.7 Hz, 1H, ArH), 7.34 (ddd, *J* = 7. 3, 4.8, 0.9 Hz, 1H, ArH), 4.04 (s, 3H, OCH_3_). The spectroscopic properties of the compound were consistent with those reported in the literature [63].

#### 4.2.3. Bpy–(imidazole)_1_ (BPI1)

The compound 1-methylimidazole (164 mg, 2.00 mmol) was dissolved in THF (10 mL) under an argon atmosphere and cooled to −78 °C. A solution of *n*-BuLi (5 mL, 0.4 M in hexane, 2.00 mmol) was added, and the mixture was stirred for 90 min. The compound 6-methoxycarbonyl-2,2**′**-bipyridine (400 mg, 1.90 mmol) was added as a solution in THF, and the color changed from colorless to purple immediately after the addition. The reaction mixture was stirred overnight at room temperature. After the reaction was quenched with H_2_O, the color of the solution turned orange. THF was removed via rotary evaporation, and the resulting light orange organic layer obtained after biphasic extraction from ethyl acetate/water was dehydrated using MgSO_4_ and concentrated to orange oil and the crude product was purified via column chromatography (neutral silica stationary phase and dichloromethane/methanol eluent), and the eluate was concentrated in vacuo to afford bpy–(imidazole)_1_ as light yellow solid. Yield: 336 mg (67%) ^1^H NMR (500 MHz, CDCl_3_) δ 8.66 (d, *J* = 4.7 Hz, 1H, ArH), 8.57 (dd, *J* = 7.9, 0.8 Hz, 1H, ArH), 8.52 (d, *J* = 8.0 Hz, 1H, ArH), 8.20 (dd, *J* = 7.7, 0.8 Hz, 1H, ArH), 7.97 (t, *J* = 7.8 Hz, 1H, ArH), 7.78 (td, *J* = 7.8, 1.8 Hz, 1H, ArH), 7.32–7.27 (m, 1H, ArH), 7.26 (s, 1H, ArH), 7.14 (s, 1H, ArH), 4.13 (s, 3H, CH_3_). ^13^C{^1^H} NMR (126 MHz, CDCl_3_) δ: 183.3, 156.2, 155.7, 154.0, 149.1, 143.0, 137.4, 137.1, 130.2, 127.4, 125.8, 124.1, 123.5, 121.9, 36.58. Anal. Calcd for C_15_H_12_N_4_O: C, 68.17, H, 4.58, N, 21.20. Found: C, 68.43, H, 4.32, N, 21.28.

#### 4.2.4. Bpy–(imidazole)_2_ (BPI2)

The compound 1-methylimidazole (410 mg, 5.00 mmol) was dissolved in THF (10 mL) under an argon atmosphere and cooled to −78 °C. A solution of *n*-BuLi (10 mL, 0.4 M in hexane, 4.00 mmol) was added, and the mixture was stirred for 90 min. The compound 6-methoxycarbonyl-2,2**′**-bipyridine (400 mg, 1.90 mmol) was added as a solution in THF, and the color changed from colorless to purple immediately after the addition. The reaction mixture was stirred overnight at room temperature. After the reaction was quenched with H_2_O, the color of the solution turned orange. THF was removed via rotary evaporation, and the resulting light orange organic layer obtained after biphasic extraction from ethyl acetate/water was dehydrated using MgSO_4_ and concentrated to orange oil and the crude product was purified via column chromatography (neutral silica stationary phase and dichloromethane/methanol eluent), and the eluate was concentrated in vacuo to afford bpy–(imidazole)_2_ as light yellow solid. Yield: 401 mg (62%). ^1^H NMR (500 MHz, CDCl_3_) δ 8.67 (d, *J* = 4.4 Hz, 1H, ArH), 8.43 (d, *J* = 7.6 Hz, 1H, ArH), 8.26 (d, *J* = 7.9 Hz, 1H, ArH), 7.89 (t, *J* = 7.7 Hz, 1H, ArH), 7.78 (t, *J* = 7.6 Hz, 1H, ArH), 7.36 (d, *J* = 7.6 Hz, 1H, ArH), 7.32–7.27 (m, 1H, ArH), 6.94 (s, 2H, ArH), 6.89 (s, 2H, ArH), 3.49 (s, 6H, CH_3_). ^13^C{^1^H} NMR (126 MHz, CDCl_3_) δ: 157.6, 155.3, 153.4, 149.3, 147.6, 138.3, 136.9, 126.8, 124.0, 123.9, 123.3, 120.9, 120.7, 75.00, 29.71. Anal. Calcd for C_19_H_18_N_6_O: C, 65.88, H, 5.24, N, 24.26. Found: C, 66.02, H, 5.29, N, 24.38.

#### 4.2.5. Complex **1**

Mn(ClO_4_)_2_·6H_2_O (144 mg, 0.400 mmol) was added to the solution of BPI1 (106 mg, 0.400 mmol) in ethanol (10 mL), and the reaction mixture was refluxed for 8 h under an N_2_ atmosphere. The reaction completion was confirmed using TLC. After the reaction, ethanol was removed via rotary evaporation, the mixture was re-dissolved in the minimum amount of acetonitrile, and the product was purified via precipitation from acetonitrile/diethyl ether. The product was crystallized using vapor diffusion of diethyl ether into a concentrated acetonitrile solution. The complex crystallizes as a 1:2 mixture of bis(aquo) and mono(aquo) ligated complexes (see discussion above and in SI). Yield: 180 mg (76%). μ_eff_ (Evans method, CD_3_CN, 25 °C): 6.04 μ_B_. Anal. Calcd for C_17_H_17.66_N_5_O_10.33_Cl_2_Mn: C, 35.01, H, 3.05, N, 12.01. Found: C, 34.64, H, 3.15, N, 11.49.

Safety Note for Complex **1**: Perchlorate salts of metal complexes such as this can potentially be shock-sensitive explosives [64]. Although we have not experienced any issues with this compound, it should only be prepared in small quantities and handled with care.

#### 4.2.6. Complex **2**

The title compound was prepared using the general method described above for complex **1**, using Mn(OTf)_2_ (90 mg, 0.25 mmol) and BPI1 (66 mg, 0.25 mmol). The product was crystallized using vapor diffusion of diethyl ether into a concentrated acetonitrile solution. Yield: 120 mg (73%). μ_eff_ (Evans method, CD_3_CN, 25 °C): 5.94 μ_B_. Anal. Calcd for C_19_H_15_N_5_O_7_S_2_F_6_Mn: C, 34.66, H, 2.30, N, 10.64. Found: C, 35.03, H, 2.61, N, 10.78.

#### 4.2.7. Complex **3**

The title compound was prepared using the general method described above for complex **1**, using MnCl_2_ (31 mg, 0.25 mmol) and BPI2 (86 mg, 0.25 mmol). The product was crystallized by vapor diffusion of diethyl ether into a concentrated ethanol solution. Yield: 75 mg (64%). μ_eff_ (Evans method, CD_3_OD, 25 °C): 6.02 μ_B_. Anal. Calcd for C_19_H_18_N_6_OCl_2_Mn: C, 48.33, H, 3.84, N, 17.80. Found: C, 48.11, H, 4.10, N, 17.71.

#### 4.2.8. Complex **4**

The title compound was prepared by the general method described above for complex **1**, using Mn(OTf)_2_ (90 mg, 0.25 mmol) and BPI2 (86 mg, 0.25 mmol). The product was crystallized by vapor diffusion of diethyl ether into a concentrated acetonitrile solution. Yield: 132 mg (75%). μ_eff_ (Evans method, CD_3_CN, 25 °C): 6.08 μ_B_. Anal. Calcd for C_21_H_18_N_6_O_7_S_2_F_6_Mn: C, 36.06, H, 2.59, N, 12.02. Found: C, 36.28, H, 2.85, N, 12.24.

### 4.3. Controlled Potential Electrolysis

Controlled potential electrolysis experiments were performed in a 50 mL bulk electrolysis cell. The cell contained an 8 cm^2^ square-plate glass carbon working electrode, an Ag/AgCl (3 M KCl) reference electrode, and a platinum wire counter electrode. Before starting the experiment, the cell was purged with dinitrogen for 30–60 min. Then, the cell was sealed, and the dioxygen concentration was measured before and during the electrolysis. During the experiment, the solutions in the electrolysis cell were vigorously stirred. The produced dioxygen was quantified in the solution using a Vernier dissolved oxygen probe. The results of the CPE with complex **4** were compared with a blank experiment in the same conditions but in the absence of a catalyst.

### 4.4. X-ray Crystallography

X-ray diffraction (XRD) studies were carried out at the Mississippi State University X-ray Facility on a Bruker AXS D8 Venture diffractometer using a three-circle goniometer in a kappa geometry with a fixed kappa angle at 54.74° and equipped with a Photon 100 CMOS active pixel sensor detector. Either monochromatized copper Kα (λ = 1.54178 Å) or molybdenum Kα (λ = 0.71703 Å) radiation was used, as specified in the Supporting Information for each structure. Crystals were mounted on a cryoloop using an oil cryoprotectant, and XRD data were collected at low temperatures (T = 100 K). All frames were integrated with the aid of the Bruker SAINT software (version 2021.4-0) using a narrow-frame algorithm. Data were corrected for absorption effects using the multi-scan method (SADABS). The structure was solved using SHELXT [65] and was refined against weighted F2 values using a full-matrix least-squares procedure with the SHELXTL-2018/3 program suite [66] as included in the APEX3 Bruker package (2019 version). Further crystallographic details for each complex can be found in the Supporting Information.

### 4.5. Density Functional Theory (DFT) Calculations

DFT calculations were carried out using Gaussian 16 (Revision C.01), [67] using default parameters unless otherwise specified. Geometry and frequency calculations were carried out starting from the crystallographic-determined coordinates when available. For calculations involving Mn^2+^, an unrestricted calculation, and *S* = 5/2 spin state were used; otherwise, restricted calculations in the singlet state were used. Counteranions are omitted. Calculations were carried out using the B3LYP functional with the D3 version of Grimme’s dispersion correction with Becke-Johnson damping (GD3BJ) and the basis set 6–311 g (d) for all atoms; a quadratically convergent procedure was used for SCF convergence [68,69]. Implicit solvation in MeCN was modeled using the polarizable continuum model and solvent-accessible surface model. Using the optimized geometry, single-point time-dependent DFT calculations were performed to determine the excited states and predict the optical spectra; for ligands, the first 20 excited states were calculated, and for complexes, the first 100 excited states were calculated. In simulated spectra, a Gaussian broadening of 0.28 eV (HWHM) was applied to approximate the broadening in the experimental spectra. For the complexes, most excited states were found to be composed of a large number of transitions between canonical molecular orbitals with no clear dominant transition; therefore, natural transition orbitals (NTOs) were calculated in Gaussian to visualize the excitations better [52].

### 4.6. Protonation and Stability Constant Determination

Protonation and binding constants were determined using potentiometric pH titration, which was carried out using a Ross 8103BN semi-micro pH probe and an Orion OrionStar T910 pH meter and automatic titrator. The pH electrode was calibrated with a 3-point buffer (Thermo Scientific, Waltham, MA, USA) set before each set of measurements. All solutions were prepared using a 0.15 *M* solution of sodium chloride in ultra-pure Milli-Q (18 MΩ∙cm) water, which was degassed by bubbling with nitrogen for 1 h to remove dissolved carbonates. Prepared solutions, titrants, and analytes were protected with a nitrogen blanket throughout the measurement process. Measurements were carried out in 6 mL samples in a jacketed cell thermostatted to 25 ± 0.5 °C for consistency.

An approximately 0.05 *M* solution of NaOH was prepared from the stock sodium chloride solution and was used as the titrant for all measurements, protected at all times with a blanket of nitrogen. This was prepared from a commercially available 50% w% aqueous solution of NaOH. Also, from the stock sodium chloride solution, an approximate 0.01 *M* solution of HCl was prepared by dilution of a stock HCl aqueous solution. The exact concentration of the NaOH titrant was determined by standardization with potassium hydrogen phthalate, determining that the NaOH solution was 0.04963 *M*. Calibration titrations with the HCl analyte solutions were performed determining the solution of HCl to be 0.0116 *M*. BPI2 and Complex **4** solutions were prepared with the HCl analyte solution at concentration of 0.001735 *M* and 0.001470 *M* respectively. The standardization titrations were analyzed with the program *pHcali* to determine the pK_w_ and to determine the Irving constants used to convert measured pH_m_ values for the titrations to p[H^+^] values that were used for fitting the equilibria in HyperQuad2013 [70,71]. Data outside the Nernstian regime (pH < 2 or >12) were excluded from analysis in all cases. Measurements were carried out in triplicate, and these data were fit simultaneously. For Complex **4**, only data below pH 8 were used for fitting log(K_Mn_); at higher pH, a brown precipitate (most likely manganese oxide) was observed to form in the analyte solution.

## Figures and Tables

**Table 1:** Association Constants Determined by Potentiometric Titration for BPI2.

Parameter	Corresponding Equilibrium	Fit Value
log(*K*_H1_)	BPI2 + H^+^ ⇌ HBPI2^+^	6.34 ± 0.09
log(*K*_H2_)	HBPI2^+^ + H^+^ ⇌ H_2_BPI2^2+^	4.60 ± 0.13
log(*K*_H3_)	H_2_BPI2^2+^ + H^+^ ⇌ H_3_BPI2^3+^	2.79 ± 0.24
log(*K*_Mn_)	BPI2 + Mn^2+^ ⇌ BPI2Mn^2+^	3.25 ± 0.45

**Table 2:** Summary of UV-Vis absorption data recorded in acetonitrile at room temperature.

	λ/nm (ε × 10^−3^/M^−1^cm^−1^) ^a^
BPI1	283(13), 299(sh)(12)
BPI2	291(8.0), 300(sh)(6.0)
**1** and **2**	287(sh)(13), 309(15), 320(sh)(14)
**3**	298(7.4), 312(sh)(5.9), 340(sh)(2.5)
**4**	300(9.0), 310(sh)(7.6), 337(sh)(1.4)

^a^ For **1** and **2**, ε values are averaged over these two spectra.

## Data Availability

CCDC 2178876–2178878 and 2301428 contain the supplementary crystallographic data for this paper. These data can be obtained free of charge via www.ccdc.cam.ac.uk/data_request/cif. All other data discussed in the paper is provided either within the article or in the Supplementary Information. Raw data files will be provided upon request.

## Supplementary Materials

The following supporting information can be downloaded at: https://www.mdpi.com/article/10.3390/molecules28207221/s1, Table S1: Summary of crystallographic data; Table S2: Selected bond lengths and angles; Table S3: Angular Distortion Parameters; Figure S1: Full asymmetric unit of Complex **1**; Figures S2–S7: ^1^H and ^13^C NMR spectra of ligand precursors and ligands; Figure S8: EPR spectra; Figures S9–S11: Orbital energy diagrams and frontier orbital pictures from DFT calculations; Figure S12: Overlaid calculated UV-Vis spectra; Figure S13: TD-DFT results for ligands; Figure S14–S15: Potentiometric titration data and speciation curves for ligand; Figure S16: CVs of complex **3** in acetonitrile with added water; Figure S17: CVs of MnCl_2_ in aqueous buffer; Figure S18: Aqueous CV scans for complex **4** at different pH’s; Figure S19: CV scans of manganese(II) perchlorate with added BPI2 ligand; Figure S20–S21: UV-Vis spectra of complexes **1** and **4** in water/acetonitrile mixtures; Figure S22: Data for controlled potential electrolysis of aqueous complex **4**; Figure S23: UV-Vis spectra before and after electrolysis; Figure S24: photograph of O_2_ bubbles; Figure S25: Expanded ESI-MS data. Zip file containing Gaussian output files for optimized geometries and TD-DFT calculations.

## Sample Availability

Samples of compounds are available from the authors.
