# Supplementary material for: Synthesis and Characterization of Bipyridyl-(Imidazole)n Mn(II) Compounds and Their Evaluation as Potential Precatalysts for Water Oxidation
Source: Molecules. 2023 Oct 23;28(20):7221. doi: 10.3390/molecules28207221 (PMC10608871; doi:10.3390/molecules28207221)

checkCIF/PLATON report

Structure factors have been supplied for datablock(s) c_sc_068_final

THIS REPORT IS FOR GUIDANCE ONLY. IF USED AS PART OF A REVIEW PROCEDURE FOR PUBLICATION, IT SHOULD NOT REPLACE THE EXPERTISE OF AN EXPERIENCED CRYSTALLOGRAPHIC REFEREE.

No syntax errors found.   CIF dictionary   Interpreting this report

Datablock: c_sc_068_final

```
Bond precision:   C-C = 0.0167 A          Wavelength=1.54178

Cell:           a=11.820(1)        b=12.7584(11)      c=13.3067(13)
                alpha=109.691(6)   beta=107.464(5)    gamma=99.058(5)
Temperature:    100 K
```

	Calculated	Reported
Volume	1726.0(3)	1726.0(3)
Space group	P 1	P 1
Hall group	P 1	P 1
Moiety formula	C17 H17 Cl Mn N5 O6, C17 H16 Cl Mn N5 O6, C17 H17 Mn N5 O3, 4(C	C51 H50 Cl6 Mn3 N15 O31
Sum formula	C51 H50 Cl6 Mn3 N15 O31	C51 H50 Cl6 Mn3 N15 O31
Mr	1746.58	1746.58
Dx,g cm-3	1.680	1.680
Z	1	1
Mu (mm-1)	7.409	7.409
F000	886.0	886.0
F000'	889.46	
h,k,lmax	14,15,16	14,15,16
Nref	14138[7069]	11220
Tmin,Tmax	0.317,0.470	0.276,0.519
Tmin'	0.154	

```
Correction method= # Reported T Limits: Tmin=0.276 Tmax=0.519
AbsCorr = MULTI-SCAN

Data completeness= 1.59/0.79        Theta(max)= 74.496
```

R(reflections)= 0.0693(9913) wR2(reflections)= 0.1757(11220)

S = 1.034 Npar= 963

The following ALERTS were generated. Each ALERT has the format
test-name_ALERT_alert-type_alert-level.
Click on the hyperlinks for more details of the test.

Alert level B

```
PLAT341_ALERT_3_B Low Bond Precision on  C-C Bonds ...............    0.01669 Ang.
PLAT354_ALERT_3_B Short  O-H (X0.82,N0.98A)  O3A      - H31A     .       0.58 Ang.
```

Alert level C

```
STRVA01_ALERT_4_C           Flack test results are ambiguous.
           From the CIF: _refine_ls_abs_structure_Flack    0.552
           From the CIF: _refine_ls_abs_structure_Flack_su    0.010
PLAT042_ALERT_1_C Calc. and Reported MoietyFormula Strings  Differ     Please Check
PLAT090_ALERT_3_C Poor Data / Parameter Ratio (Zmax > 18) ........       7.17 Note
PLAT222_ALERT_3_C NonSolvent Resd 1  H   Uiso(max)/Uiso(min) Range       4.1 Ratio
PLAT241_ALERT_2_C High   'MainMol' Ueq as Compared to Neighbors of       O61A Check
PLAT250_ALERT_2_C Large U3/U1 Ratio for Average U(i,j) Tensor ....        2.5 Note
PLAT250_ALERT_2_C Large U3/U1 Ratio for Average U(i,j) Tensor ....        2.9 Note
PLAT250_ALERT_2_C Large U3/U1 Ratio for Average U(i,j) Tensor ....        3.1 Note
PLAT354_ALERT_3_C Short  O-H (X0.82,N0.98A)  O2C      - H21C     .       0.65 Ang.
PLAT355_ALERT_3_C Long   O-H (X0.82,N0.98A)  O2B      - H22B     .       1.06 Ang.
PLAT369_ALERT_2_C Long   C(sp2)-C(sp2) Bond  C5B      - C6B      .       1.53 Ang.
PLAT369_ALERT_2_C Long   C(sp2)-C(sp2) Bond  C10C     - C11C     .       1.54 Ang.
PLAT415_ALERT_2_C Short Inter D-H..H-X       H4A      ..H22B     .       2.03 Ang.
                                                 x,1+y,z  =      1_565 Check
PLAT751_ALERT_4_C Bond    Calc     0.89000, Rep    0.889(7) ......  Senseless s.u.
              O2A      -H21A           1_555   1_555 ........     #   18 Check
PLAT751_ALERT_4_C Bond    Calc     0.58000, Rep    0.583(9) ......  Senseless s.u.
              O3A      -H31A           1_555   1_555 ........     #   19 Check
PLAT751_ALERT_4_C Bond    Calc     0.81000, Rep    0.805(8) ......  Senseless s.u.
              O2B      -H21B           1_555   1_555 ........     #   65 Check
PLAT751_ALERT_4_C Bond    Calc     1.06000, Rep    1.065(8) ......  Senseless s.u.
              O2B      -H22B           1_555   1_555 ........     #   66 Check
PLAT751_ALERT_4_C Bond    Calc     0.65000, Rep    0.653(7) ......  Senseless s.u.
              O2C      -H21C           1_555   1_555 ........     #  112 Check
PLAT752_ALERT_4_C Angle   Calc      113.00, Rep    112.9(7) ......  Senseless s.u.
              MN1A    -O2A     -H21A       1_555  1_555  1_555     #   29 Check
PLAT752_ALERT_4_C Angle   Calc      129.00, Rep   128.6(12) ......  Senseless s.u.
              MN1A    -O3A     -H31A       1_555  1_555  1_555     #   30 Check
PLAT752_ALERT_4_C Angle   Calc      129.00, Rep    128.7(7) ......  Senseless s.u.
              MN1B    -O2B     -H21B       1_555  1_555  1_555     #  114 Check
PLAT752_ALERT_4_C Angle   Calc      129.00, Rep    129.4(6) ......  Senseless s.u.
              MN1B    -O2B     -H22B       1_555  1_555  1_555     #  115 Check
PLAT752_ALERT_4_C Angle   Calc       98.00, Rep     98.4(8) ......  Senseless s.u.
              H21B    -O2B     -H22B       1_555  1_555  1_555     #  116 Check
PLAT752_ALERT_4_C Angle   Calc      100.00, Rep   100.3(10) ......  Senseless s.u.
              MN1C    -O2C     -H21C       1_555  1_555  1_555     #  200 Check
PLAT907_ALERT_2_C Flack x > 0.5, Structure Needs to be Inverted? .       0.55 Check
PLAT911_ALERT_3_C Missing FCF Refl Between Thmin & STh/L=    0.600        101 Report
```

```
PLAT915_ALERT_3_C No Flack x Check Done: Low Friedel Pair Coverage        61 %
```

Alert level G

```
PLAT007_ALERT_5_G Number of Unrefined Donor-H Atoms .............          5 Report
PLAT033_ALERT_4_G Flack x Value Deviates > 3.0 * sigma from Zero .      0.552 Note
PLAT083_ALERT_2_G SHELXL Second Parameter in WGHT  Unusually Large       6.49 Why ?
PLAT242_ALERT_2_G Low    'MainMol' Ueq as Compared to Neighbors of       Cl2A Check
PLAT242_ALERT_2_G Low    'MainMol' Ueq as Compared to Neighbors of       Cl6A Check
PLAT244_ALERT_4_G Low    'Solvent' Ueq as Compared to Neighbors of       Cl1A Check
PLAT244_ALERT_4_G Low    'Solvent' Ueq as Compared to Neighbors of       Cl3A Check
PLAT244_ALERT_4_G Low    'Solvent' Ueq as Compared to Neighbors of       Cl4A Check
PLAT244_ALERT_4_G Low    'Solvent' Ueq as Compared to Neighbors of       Cl5A Check
PLAT432_ALERT_2_G Short Inter X...Y Contact  O11A     ..C11A     .       2.91 Ang.
                                                     x,y,z  =      1_555 Check
PLAT432_ALERT_2_G Short Inter X...Y Contact  O24A     ..C1A      .       2.98 Ang.
                                                     x,y,z  =      1_555 Check
PLAT432_ALERT_2_G Short Inter X...Y Contact  O41A     ..C11B     .       2.87 Ang.
                                                     x,y,z  =      1_555 Check
PLAT432_ALERT_2_G Short Inter X...Y Contact  O41A     ..C10B     .       2.93 Ang.
                                                     x,y,z  =      1_555 Check
PLAT432_ALERT_2_G Short Inter X...Y Contact  O64A     ..C11A     .       2.94 Ang.
                                                x,-1+y,1+z  =      1_546 Check
PLAT720_ALERT_4_G Number of Unusual/Non-Standard Labels .........          4 Note
PLAT794_ALERT_5_G Tentative Bond Valency for Mn1A      (II)      .       2.25 Info
PLAT794_ALERT_5_G Tentative Bond Valency for Mn1B      (II)      .       2.15 Info
PLAT794_ALERT_5_G Tentative Bond Valency for Mn1C      (II)      .       2.25 Info
PLAT910_ALERT_3_G Missing # of FCF Reflection(s) Below Theta(Min).          1 Note
PLAT912_ALERT_4_G Missing # of FCF Reflections Above STh/L=  0.600         63 Note
PLAT933_ALERT_2_G Number of HKL-OMIT Records in Embedded .res File          4 Note
PLAT965_ALERT_2_G The SHELXL WEIGHT Optimisation has not Converged     Please Check
PLAT978_ALERT_2_G Number C-C Bonds with Positive Residual Density.          0 Info
PLAT992_ALERT_5_G Repd & Actual _reflns_number_gt Values Differ by          4 Check
```

```
   0 ALERT level A = Most likely a serious problem - resolve or explain
   2 ALERT level B = A potentially serious problem, consider carefully
  27 ALERT level C = Check. Ensure it is not caused by an omission or oversight
  24 ALERT level G = General information/check it is not something unexpected

   1 ALERT type 1 CIF construction/syntax error, inconsistent or missing data
  19 ALERT type 2 Indicator that the structure model may be wrong or deficient
   9 ALERT type 3 Indicator that the structure quality may be low
  19 ALERT type 4 Improvement, methodology, query or suggestion
   5 ALERT type 5 Informative message, check
```

---

checkCIF publication errors

Alert level A

```
PUBL004_ALERT_1_A  The contact author's name and address are missing,
            _publ_contact_author_name and _publ_contact_author_address.
PUBL005_ALERT_1_A  _publ_contact_author_email, _publ_contact_author_fax and
            _publ_contact_author_phone are all missing.
```

```
            At least one of these should be present.
PUBL006_ALERT_1_A  _publ_requested_journal is missing
            e.g. 'Acta Crystallographica Section C'
PUBL008_ALERT_1_A  _publ_section_title is missing. Title of paper.
PUBL009_ALERT_1_A  _publ_author_name is missing. List of author(s) name(s).
PUBL010_ALERT_1_A  _publ_author_address is missing. Author(s) address(es).
PUBL012_ALERT_1_A  _publ_section_abstract is missing.
            Abstract of paper in English.
```

Alert level G

```
PUBL017_ALERT_1_G The _publ_section_references section is missing or
            empty.
```

---

   7 **ALERT level A** = Data missing that is essential or data in wrong format
   1 **ALERT level G** = General alerts. Data that may be required is missing

---

Publication of your CIF

You should attempt to resolve as many as possible of the alerts in all categories. Often the minor alerts point to easily fixed oversights, errors and omissions in your CIF or refinement strategy, so attention to these fine details can be worthwhile. In order to resolve some of the more serious problems it may be necessary to carry out additional measurements or structure refinements. However, the nature of your study may justify the reported deviations from journal submission requirements and the more serious of these should be commented upon in the discussion or experimental section of a paper or in the "special_details" fields of the CIF. *checkCIF* was carefully designed to identify outliers and unusual parameters, but every test has its limitations and alerts that are not important in a particular case may appear. Conversely, the absence of alerts does not guarantee there are no aspects of the results needing attention. It is up to the individual to critically assess their own results and, if necessary, seek expert advice.

If level A alerts remain, which you believe to be justified deviations, and you intend to submit this CIF for publication in a journal, you should additionally insert an explanation in your CIF using the Validation Reply Form (VRF) below. This will allow your explanation to be considered as part of the review process.

Validation response form

Please find below a validation response form (VRF) that can be filled in and pasted into your CIF.

```
# start Validation Reply Form
_vrf_PUBL004_GLOBAL
;
PROBLEM: The contact author's name and address are missing,
RESPONSE: ...
;
_vrf_PUBL005_GLOBAL
;
PROBLEM: _publ_contact_author_email, _publ_contact_author_fax and
RESPONSE: ...
```

```
;
_vrf_PUBL006_GLOBAL
;
PROBLEM: _publ_requested_journal is missing
RESPONSE: ...
;
_vrf_PUBL008_GLOBAL
;
PROBLEM: _publ_section_title is missing. Title of paper.
RESPONSE: ...
;
_vrf_PUBL009_GLOBAL
;
PROBLEM: _publ_author_name is missing. List of author(s) name(s).
RESPONSE: ...
;
_vrf_PUBL010_GLOBAL
;
PROBLEM: _publ_author_address is missing. Author(s) address(es).
RESPONSE: ...
;
_vrf_PUBL012_GLOBAL
;
PROBLEM: _publ_section_abstract is missing.
RESPONSE: ...
;
_vrf_STRVA01_c_sc_068_final
;
PROBLEM: Flack test results are ambiguous.
RESPONSE: ...
;
_vrf_PLAT341_c_sc_068_final
;
PROBLEM: Low Bond Precision on  C-C Bonds ..............    0.01669 Ang.
RESPONSE: ...
;
_vrf_PLAT354_c_sc_068_final
;
PROBLEM: Short  O-H (X0.82,N0.98A)  O3A      - H31A     .       0.58 Ang.
RESPONSE: ...
;
_vrf_PLAT042_c_sc_068_final
;
PROBLEM: Calc. and Reported MoietyFormula Strings  Differ     Please Check
RESPONSE: ...
;
_vrf_PLAT090_c_sc_068_final
;
PROBLEM: Poor Data / Parameter Ratio (Zmax > 18) ........       7.17 Note
RESPONSE: ...
;
_vrf_PLAT222_c_sc_068_final
;
PROBLEM: NonSolvent Resd 1  H   Uiso(max)/Uiso(min) Range        4.1 Ratio
RESPONSE: ...
;
_vrf_PLAT241_c_sc_068_final
```

```
;
PROBLEM: High   'MainMol' Ueq as Compared to Neighbors of       O61A Check
RESPONSE: ...
;
_vrf_PLAT250_c_sc_068_final
;
PROBLEM: Large U3/U1 Ratio for Average U(i,j) Tensor ....        2.5 Note
RESPONSE: ...
;
_vrf_PLAT355_c_sc_068_final
;
PROBLEM: Long   O-H (X0.82,N0.98A)  O2B      - H22B     .       1.06 Ang.
RESPONSE: ...
;
_vrf_PLAT369_c_sc_068_final
;
PROBLEM: Long   C(sp2)-C(sp2) Bond  C5B      - C6B      .       1.53 Ang.
RESPONSE: ...
;
_vrf_PLAT415_c_sc_068_final
;
PROBLEM: Short Inter D-H..H-X       H4A      ..H22B     .       2.03 Ang.
RESPONSE: ...
;
_vrf_PLAT751_c_sc_068_final
;
PROBLEM: Bond    Calc     0.89000, Rep    0.889(7) ......  Senseless s.u.
RESPONSE: ...
;
_vrf_PLAT752_c_sc_068_final
;
PROBLEM: Angle   Calc      113.00, Rep    112.9(7) ......  Senseless s.u.
RESPONSE: ...
;
_vrf_PLAT907_c_sc_068_final
;
PROBLEM: Flack x > 0.5, Structure Needs to be Inverted? .       0.55 Check
RESPONSE: ...
;
_vrf_PLAT911_c_sc_068_final
;
PROBLEM: Missing FCF Refl Between Thmin & STh/L=    0.600        101 Report
RESPONSE: ...
;
_vrf_PLAT915_c_sc_068_final
;
PROBLEM: No Flack x Check Done: Low Friedel Pair Coverage         61 %
RESPONSE: ...
;
# end Validation Reply Form
```

If you wish to submit your CIF for publication in Acta Crystallographica Section C or E, you should upload your CIF via the web. If you wish to submit your CIF for publication in IUCrData you should upload your CIF via the web. If your CIF is to form part of a submission to another IUCr journal, you will be asked, either during electronic submission or by the Co-editor handling your paper, to upload your CIF via our web site.

PLATON version of 06/07/2023; check.def file version of 30/06/2023

Datablock c_sc_068_final - ellipsoid plot

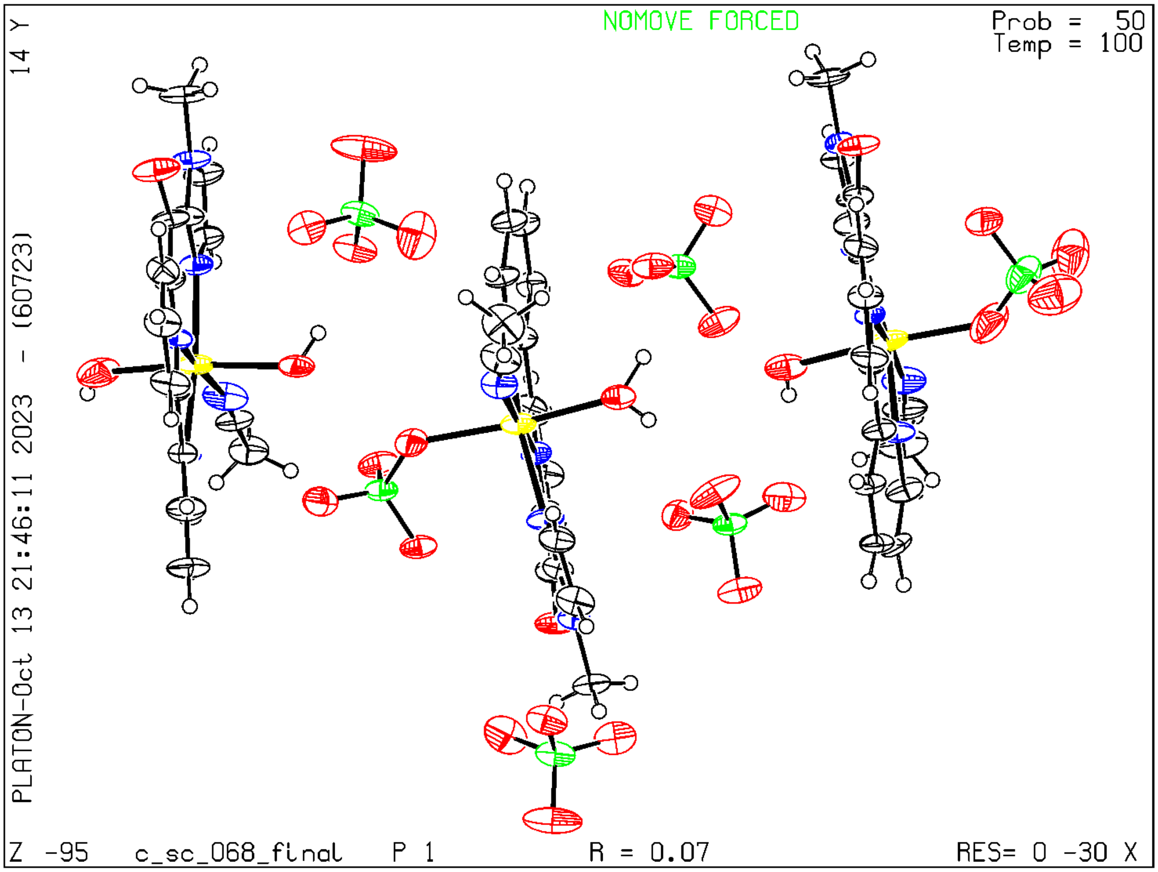

Supplement: Supplementary file 1 [file molecules-28-07221-s001.zip › crystal_data_for_resubmission/checkcif-complex1-revised.pdf]
