# Supplementary material for: Synthesis and Characterization of Bipyridyl-(Imidazole)n Mn(II) Compounds and Their Evaluation as Potential Precatalysts for Water Oxidation
Source: Molecules. 2023 Oct 23;28(20):7221. doi: 10.3390/molecules28207221 (PMC10608871; doi:10.3390/molecules28207221)

checkCIF/PLATON report

Structure factors have been supplied for datablock(s) sc001_rev

THIS REPORT IS FOR GUIDANCE ONLY. IF USED AS PART OF A REVIEW PROCEDURE FOR PUBLICATION, IT SHOULD NOT REPLACE THE EXPERTISE OF AN EXPERIENCED CRYSTALLOGRAPHIC REFEREE.

No syntax errors found. CIF dictionary Interpreting this report

Datablock: sc001_rev

```
Bond precision:   C-C = 0.0052 A          Wavelength=1.54178

Cell:              a=8.0555(2)     b=10.3178(2)        c=29.9387(6)
                   alpha=90        beta=93.604(1)      gamma=90
Temperature:       200 K

                 Calculated                         Reported
Volume           2483.44(9)                         2483.44(9)
Space group      P 21/c                             P 21/c
Hall group       -P 2ybc                            -P 2ybc
Moiety formula   C19 H15 F6 Mn N5 O7 S2             C19 H15 F6 Mn N5 O7 S2
Sum formula      C19 H15 F6 Mn N5 O7 S2             C19 H15 F6 Mn N5 O7 S2
Mr               658.42                             658.42
Dx,g cm-3        1.761                              1.761
Z                4                                  4
Mu (mm-1)        6.803                              6.803
F000             1324.0                             1324.0
F000'            1329.56
h,k,lmax         10,12,37                           10,12,37
Nref             5077                               5065
Tmin,Tmax        0.512,0.827                        0.236,0.832
Tmin'            0.125

Correction method= # Reported T Limits: Tmin=0.236 Tmax=0.832
AbsCorr = MULTI-SCAN

Data completeness= 0.998            Theta(max)= 74.487

                                                        wR2(reflections)=
R(reflections)= 0.0520( 4164)                           0.1068( 5065)

S = 1.093                    Npar= 400
```

The following ALERTS were generated. Each ALERT has the format
test-name_ALERT_alert-type_alert-level.
Click on the hyperlinks for more details of the test.

Alert level C

```
PLAT242_ALERT_2_C Low    'MainMol' Ueq as Compared to Neighbors of         S1 Check
PLAT242_ALERT_2_C Low    'MainMol' Ueq as Compared to Neighbors of         S2 Check
PLAT906_ALERT_3_C Large K Value in the Analysis of Variance ......      2.747 Check
PLAT911_ALERT_3_C Missing FCF Refl Between Thmin & STh/L=    0.600          7 Report
```

Alert level G

```
PLAT002_ALERT_2_G Number of Distance or Angle Restraints on AtSite         12 Note
PLAT003_ALERT_2_G Number of Uiso or Uij Restrained non-H Atoms ...          8 Report
PLAT083_ALERT_2_G SHELXL Second Parameter in WGHT  Unusually Large       5.43 Why ?
PLAT175_ALERT_4_G The CIF-Embedded .res File Contains SAME Records          2 Report
PLAT177_ALERT_4_G The CIF-Embedded .res File Contains DELU Records          1 Report
PLAT178_ALERT_4_G The CIF-Embedded .res File Contains SIMU Records          1 Report
PLAT188_ALERT_3_G A Non-default SIMU Restraint Value has been used     0.0050 Report
PLAT189_ALERT_3_G A Non-default SAME Restraint Value for First Par     0.0050 Report
PLAT189_ALERT_3_G A Non-default SAME Restraint Value for SecondPar     0.0400 Report
PLAT189_ALERT_3_G A Non-default SAME Restraint Value for First Par     0.0050 Report
PLAT189_ALERT_3_G A Non-default SAME Restraint Value for SecondPar     0.0400 Report
PLAT192_ALERT_3_G A Non-default DELU Restraint Value for First Par     0.0050 Report
PLAT192_ALERT_3_G A Non-default DELU Restraint Value for SecondPar     0.0050 Report
PLAT242_ALERT_2_G Low    'MainMol' Ueq as Compared to Neighbors of        C19 Check
PLAT300_ALERT_4_G Atom Site Occupancy of F1A       Constrained at         0.5 Check
PLAT300_ALERT_4_G Atom Site Occupancy of F1B       Constrained at         0.5 Check
PLAT300_ALERT_4_G Atom Site Occupancy of F2A       Constrained at         0.5 Check
PLAT300_ALERT_4_G Atom Site Occupancy of F2B       Constrained at         0.5 Check
PLAT300_ALERT_4_G Atom Site Occupancy of F3A       Constrained at         0.5 Check
PLAT300_ALERT_4_G Atom Site Occupancy of F3B       Constrained at         0.5 Check
PLAT300_ALERT_4_G Atom Site Occupancy of C18A      Constrained at         0.5 Check
PLAT300_ALERT_4_G Atom Site Occupancy of C18B      Constrained at         0.5 Check
PLAT301_ALERT_3_G Main Residue  Disorder ..............(Resd  1  )        10% Note
PLAT794_ALERT_5_G Tentative Bond Valency for Mn1       (II)      .       2.15 Info
PLAT860_ALERT_3_G Number of Least-Squares Restraints ............         138 Note
PLAT912_ALERT_4_G Missing # of FCF Reflections Above STh/L=  0.600          6 Note
PLAT933_ALERT_2_G Number of HKL-OMIT Records in Embedded .res File          9 Note
PLAT978_ALERT_2_G Number C-C Bonds with Positive Residual Density.          0 Info
```

0 **ALERT level A** = Most likely a serious problem - resolve or explain
0 **ALERT level B** = A potentially serious problem, consider carefully
4 **ALERT level C** = Check. Ensure it is not caused by an omission or oversight
28 **ALERT level G** = General information/check it is not something unexpected

0 ALERT type 1 CIF construction/syntax error, inconsistent or missing data
8 ALERT type 2 Indicator that the structure model may be wrong or deficient
11 ALERT type 3 Indicator that the structure quality may be low
12 ALERT type 4 Improvement, methodology, query or suggestion
1 ALERT type 5 Informative message, check

checkCIF publication errors

Alert level A

```
PUBL004_ALERT_1_A  The contact author's name and address are missing,
            _publ_contact_author_name and _publ_contact_author_address.
PUBL005_ALERT_1_A  _publ_contact_author_email, _publ_contact_author_fax and
            _publ_contact_author_phone are all missing.
            At least one of these should be present.
PUBL006_ALERT_1_A  _publ_requested_journal is missing
            e.g. 'Acta Crystallographica Section C'
PUBL008_ALERT_1_A  _publ_section_title is missing. Title of paper.
PUBL009_ALERT_1_A  _publ_author_name is missing. List of author(s) name(s).
PUBL010_ALERT_1_A  _publ_author_address is missing. Author(s) address(es).
PUBL012_ALERT_1_A  _publ_section_abstract is missing.
            Abstract of paper in English.
```

Alert level G

```
PUBL017_ALERT_1_G The _publ_section_references section is missing or
            empty.
```

---

7 **ALERT level A** = Data missing that is essential or data in wrong format
1 **ALERT level G** = General alerts. Data that may be required is missing

---

Publication of your CIF

You should attempt to resolve as many as possible of the alerts in all categories. Often the minor alerts point to easily fixed oversights, errors and omissions in your CIF or refinement strategy, so attention to these fine details can be worthwhile. In order to resolve some of the more serious problems it may be necessary to carry out additional measurements or structure refinements. However, the nature of your study may justify the reported deviations from journal submission requirements and the more serious of these should be commented upon in the discussion or experimental section of a paper or in the "special_details" fields of the CIF. *checkCIF* was carefully designed to identify outliers and unusual parameters, but every test has its limitations and alerts that are not important in a particular case may appear. Conversely, the absence of alerts does not guarantee there are no aspects of the results needing attention. It is up to the individual to critically assess their own results and, if necessary, seek expert advice.

If level A alerts remain, which you believe to be justified deviations, and you intend to submit this CIF for publication in a journal, you should additionally insert an explanation in your CIF using the Validation Reply Form (VRF) below. This will allow your explanation to be considered as part of the review process.

Validation response form

Please find below a validation response form (VRF) that can be filled in and pasted into your CIF.

```
# start Validation Reply Form
_vrf_PUBL004_GLOBAL
;
PROBLEM: The contact author's name and address are missing,
RESPONSE: ...
;
_vrf_PUBL005_GLOBAL
;
PROBLEM: _publ_contact_author_email, _publ_contact_author_fax and
RESPONSE: ...
;
_vrf_PUBL006_GLOBAL
;
PROBLEM: _publ_requested_journal is missing
RESPONSE: ...
;
_vrf_PUBL008_GLOBAL
;
PROBLEM: _publ_section_title is missing. Title of paper.
RESPONSE: ...
;
_vrf_PUBL009_GLOBAL
;
PROBLEM: _publ_author_name is missing. List of author(s) name(s).
RESPONSE: ...
;
_vrf_PUBL010_GLOBAL
;
PROBLEM: _publ_author_address is missing. Author(s) address(es).
RESPONSE: ...
;
_vrf_PUBL012_GLOBAL
;
PROBLEM: _publ_section_abstract is missing.
RESPONSE: ...
;
_vrf_PLAT242_sc001_rev
;
PROBLEM: Low    'MainMol' Ueq as Compared to Neighbors of         S1 Check
RESPONSE: ...
;
_vrf_PLAT906_sc001_rev
;
PROBLEM: Large K Value in the Analysis of Variance ......      2.747 Check
RESPONSE: ...
;
_vrf_PLAT911_sc001_rev
;
PROBLEM: Missing FCF Refl Between Thmin & STh/L=    0.600          7 Report
RESPONSE: ...
;
# end Validation Reply Form
```

If you wish to submit your CIF for publication in Acta Crystallographica Section C or E, you should upload your CIF via the web. If you wish to submit your CIF for publication in IUCrData you should upload your CIF via the web. If your CIF is to form part of a submission to another IUCr journal, you will be asked, either during electronic submission or by the Co-editor handling your paper, to upload your CIF via our web site.

PLATON version of 06/07/2023; check.def file version of 30/06/2023

Datablock sc001_rev - ellipsoid plot

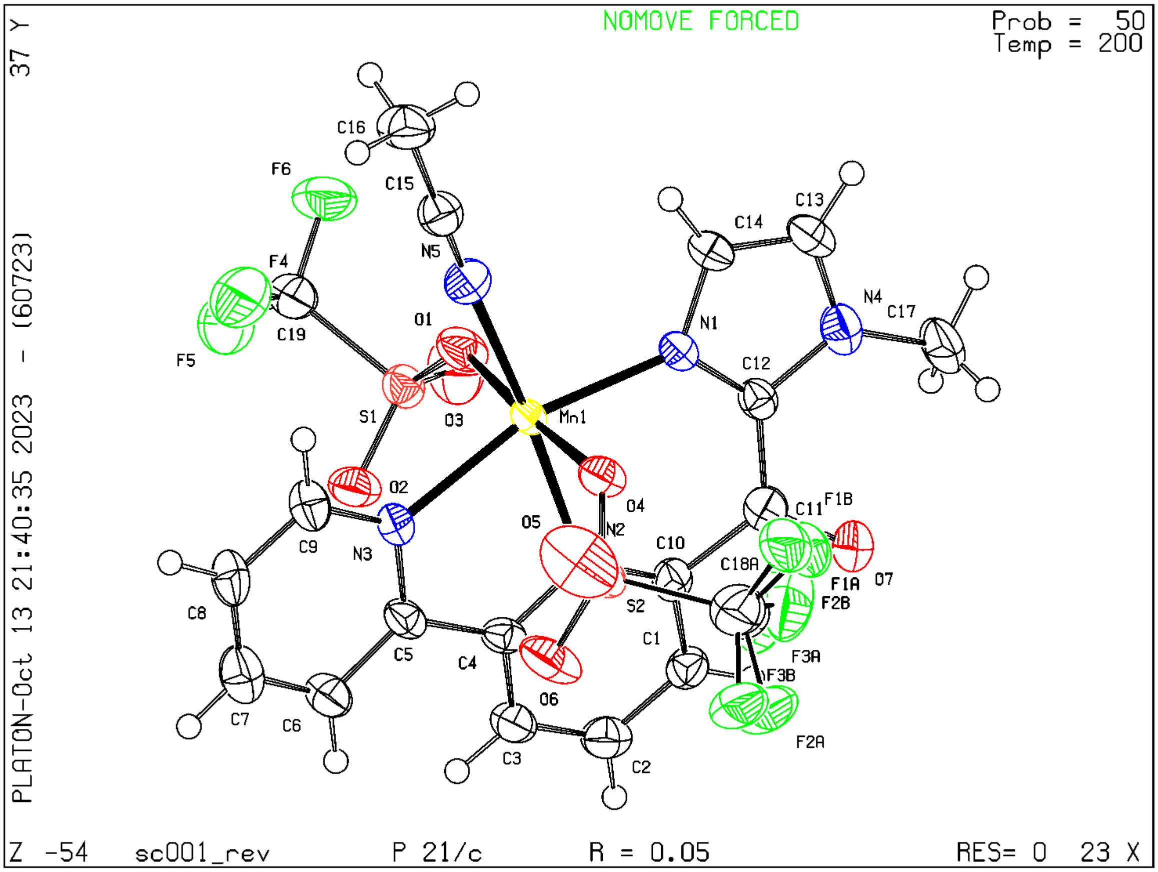

Supplement: Supplementary file 1 [file molecules-28-07221-s001.zip › crystal_data_for_resubmission/checkcif-complex2-revised.pdf]
