# Supplementary material for: Synthesis and Characterization of Bipyridyl-(Imidazole)n Mn(II) Compounds and Their Evaluation as Potential Precatalysts for Water Oxidation
Source: Molecules. 2023 Oct 23;28(20):7221. doi: 10.3390/molecules28207221 (PMC10608871; doi:10.3390/molecules28207221)

checkCIF/PLATON report

Structure factors have been supplied for datablock(s) Cu_C_SC_044_Final

THIS REPORT IS FOR GUIDANCE ONLY. IF USED AS PART OF A REVIEW PROCEDURE FOR PUBLICATION, IT SHOULD NOT REPLACE THE EXPERTISE OF AN EXPERIENCED CRYSTALLOGRAPHIC REFEREE.

No syntax errors found.   CIF dictionary   Interpreting this report

Datablock: Cu_C_SC_044_Final

```
Bond precision:  C-C = 0.0036 A          Wavelength=1.54178

Cell:            a=19.775(5)     b=17.494(4)        c=14.574(4)
                 alpha=90        beta=129.016(8)    gamma=90
Temperature:     160 K

                Calculated                Reported
Volume          3917.3(18)                3917.3(17)
Space group     C 2/c                     C 2/c
Hall group      -C 2yc                    -C 2yc
Moiety formula  C19 H18 Cl2 Mn N6 O       C19 H18 Cl2 Mn N6 O
Sum formula     C19 H18 Cl2 Mn N6 O       C19 H18 Cl2 Mn N6 O
Mr              472.23                    472.23
Dx,g cm-3       1.602                     1.601
Z               8                         8
Mu (mm-1)       8.198                     8.198
F000            1928.0                    1928.0
F000'           1934.11
h,k,lmax        25,22,18                  25,22,18
Nref            4292                      4230
Tmin,Tmax       0.155,0.371               0.176,0.437
Tmin'           0.046

Correction method= # Reported T Limits: Tmin=0.176 Tmax=0.437
AbsCorr = MULTI-SCAN

Data completeness= 0.986          Theta(max)= 80.305

R(reflections)= 0.0374( 3698)     wR2(reflections)= 0.0943( 4230)

S = 1.035                   Npar= 266
```

The following ALERTS were generated. Each ALERT has the format
test-name_ALERT_alert-type_alert-level.
Click on the hyperlinks for more details of the test.

Alert level C

```
PLAT094_ALERT_2_C Ratio of Maximum / Minimum Residual Density ....       2.85 Report
PLAT911_ALERT_3_C Missing FCF Refl Between Thmin & STh/L=    0.600          2 Report
```

Alert level G

```
PLAT007_ALERT_5_G Number of Unrefined Donor-H Atoms ..............          1 Report
PLAT083_ALERT_2_G SHELXL Second Parameter in WGHT  Unusually Large       8.27 Why ?
PLAT128_ALERT_4_G Alternate Setting for Input Space Group     C2/c       I2/a Note
PLAT232_ALERT_2_G Hirshfeld Test Diff (M-X)  Mn1      --Cl2      .        6.5 s.u.
PLAT794_ALERT_5_G Tentative Bond Valency for Mn1       (II)      .       1.92 Info
PLAT883_ALERT_1_G No Info/Value for _atom_sites_solution_primary .     Please Do !
PLAT912_ALERT_4_G Missing # of FCF Reflections Above STh/L=  0.600         60 Note
PLAT965_ALERT_2_G The SHELXL WEIGHT Optimisation has not Converged     Please Check
PLAT978_ALERT_2_G Number C-C Bonds with Positive Residual Density.          9 Info
```

0 **ALERT level A** = Most likely a serious problem - resolve or explain
0 **ALERT level B** = A potentially serious problem, consider carefully
2 **ALERT level C** = Check. Ensure it is not caused by an omission or oversight
9 **ALERT level G** = General information/check it is not something unexpected

1 ALERT type 1 CIF construction/syntax error, inconsistent or missing data
5 ALERT type 2 Indicator that the structure model may be wrong or deficient
1 ALERT type 3 Indicator that the structure quality may be low
2 ALERT type 4 Improvement, methodology, query or suggestion
2 ALERT type 5 Informative message, check

checkCIF publication errors

Alert level A

```
PUBL004_ALERT_1_A  The contact author's name and address are missing,
            _publ_contact_author_name and _publ_contact_author_address.
PUBL005_ALERT_1_A  _publ_contact_author_email, _publ_contact_author_fax and
            _publ_contact_author_phone are all missing.
            At least one of these should be present.
PUBL006_ALERT_1_A  _publ_requested_journal is missing
            e.g. 'Acta Crystallographica Section C'
PUBL008_ALERT_1_A  _publ_section_title is missing. Title of paper.
PUBL009_ALERT_1_A  _publ_author_name is missing. List of author(s) name(s).
PUBL010_ALERT_1_A  _publ_author_address is missing. Author(s) address(es).
PUBL012_ALERT_1_A  _publ_section_abstract is missing.
            Abstract of paper in English.
```

Alert level G

```
PUBL017_ALERT_1_G The _publ_section_references section is missing or
            empty.
```

7 **ALERT level A** = Data missing that is essential or data in wrong format
1 **ALERT level G** = General alerts. Data that may be required is missing

Publication of your CIF

You should attempt to resolve as many as possible of the alerts in all categories. Often the minor alerts point to easily fixed oversights, errors and omissions in your CIF or refinement strategy, so attention to these fine details can be worthwhile. In order to resolve some of the more serious problems it may be necessary to carry out additional measurements or structure refinements. However, the nature of your study may justify the reported deviations from journal submission requirements and the more serious of these should be commented upon in the discussion or experimental section of a paper or in the "special_details" fields of the CIF. *checkCIF* was carefully designed to identify outliers and unusual parameters, but every test has its limitations and alerts that are not important in a particular case may appear. Conversely, the absence of alerts does not guarantee there are no aspects of the results needing attention. It is up to the individual to critically assess their own results and, if necessary, seek expert advice.

If level A alerts remain, which you believe to be justified deviations, and you intend to submit this CIF for publication in a journal, you should additionally insert an explanation in your CIF using the Validation Reply Form (VRF) below. This will allow your explanation to be considered as part of the review process.

Validation response form

Please find below a validation response form (VRF) that can be filled in and pasted into your CIF.

```
# start Validation Reply Form
_vrf_PUBL004_GLOBAL
;
PROBLEM: The contact author’s name and address are missing,
RESPONSE: ...
;
_vrf_PUBL005_GLOBAL
;
PROBLEM: _publ_contact_author_email, _publ_contact_author_fax and
RESPONSE: ...
;
_vrf_PUBL006_GLOBAL
;
PROBLEM: _publ_requested_journal is missing
RESPONSE: ...
;
_vrf_PUBL008_GLOBAL
;
PROBLEM: _publ_section_title is missing. Title of paper.
RESPONSE: ...
;
_vrf_PUBL009_GLOBAL
;
PROBLEM: _publ_author_name is missing. List of author(s) name(s).
RESPONSE: ...
;
_vrf_PUBL010_GLOBAL
;
PROBLEM: _publ_author_address is missing. Author(s) address(es).
RESPONSE: ...
;
_vrf_PUBL012_GLOBAL
;
PROBLEM: _publ_section_abstract is missing.
```

```
RESPONSE: ...
;
_vrf_PLAT094_Cu_C_SC_044_Final
;
PROBLEM: Ratio of Maximum / Minimum Residual Density ....       2.85 Report
RESPONSE: ...
;
_vrf_PLAT911_Cu_C_SC_044_Final
;
PROBLEM: Missing FCF Refl Between Thmin & STh/L=    0.600           2 Report
RESPONSE: ...
;
# end Validation Reply Form
```

If you wish to submit your CIF for publication in Acta Crystallographica Section C or E, you should upload your CIF via the web. If you wish to submit your CIF for publication in IUCrData you should upload your CIF via the web. If your CIF is to form part of a submission to another IUCr journal, you will be asked, either during electronic submission or by the Co-editor handling your paper, to upload your CIF via our web site.

PLATON version of 13/07/2021; check.def file version of 13/07/2021

Datablock Cu_C_SC_044_Final - ellipsoid plot

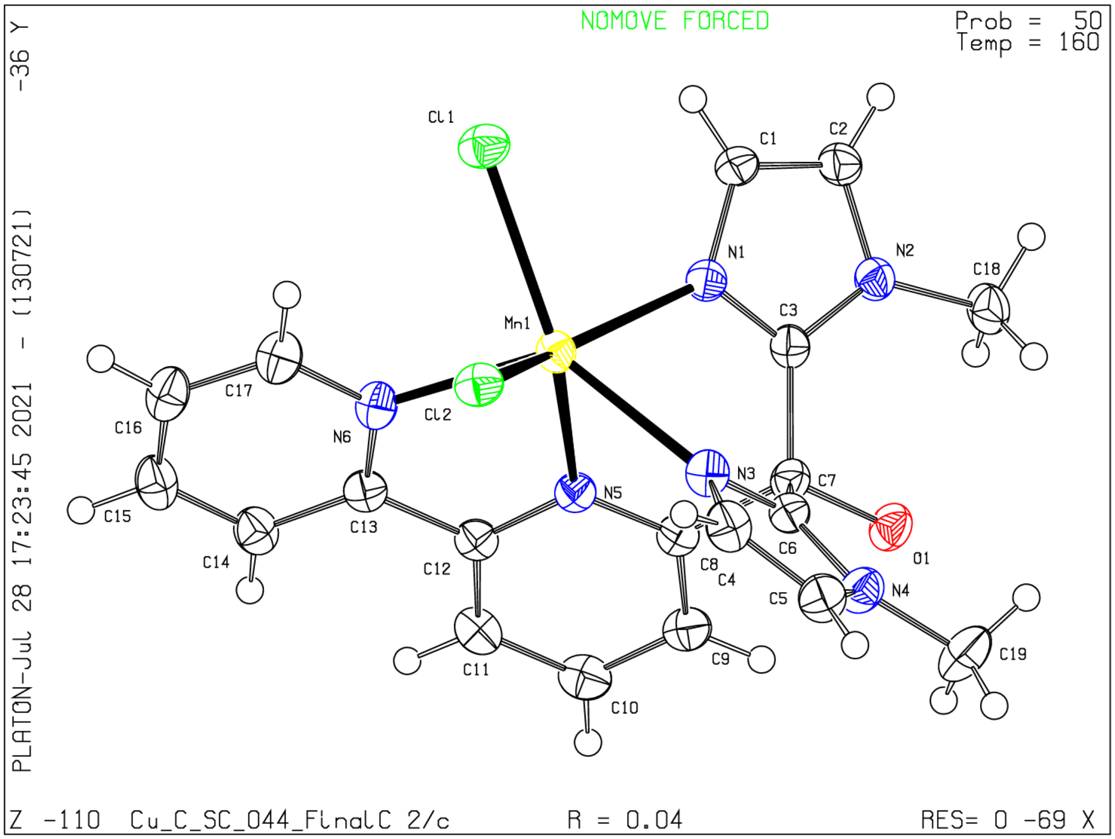

Supplement: Supplementary file 1 [file molecules-28-07221-s001.zip › crystal_data_for_resubmission/compound3_checkcif.pdf]
