# Supplementary material for: Synthesis and Characterization of Bipyridyl-(Imidazole)n Mn(II) Compounds and Their Evaluation as Potential Precatalysts for Water Oxidation
Source: Molecules. 2023 Oct 23;28(20):7221. doi: 10.3390/molecules28207221 (PMC10608871; doi:10.3390/molecules28207221)
Supplement: Supplementary file 1 [file molecules-28-07221-s001.zip › Supporting information_molecules_revised.pdf]

Supporting Information

for

Synthesis and Characterization of Bipyridyl-(Imidazole)$_n$ Mn(II) Compounds and their Evaluation as Potential Precatalysts for Water Oxidation

Ge Mu, Ryan B. Gaynor, Baylee N. McIntyre, Bruno Donnadieu, and Sidney E. Creutz*

Department of Chemistry, Mississippi State University, Mississippi State, MS USA 39762

Email: screutz@chemistry.msstate.edu

Contents

Index	*Page*
X-ray crystallographic summary tables	S2
Table of selected bond lengths and angles for complexes	S3
NMR spectra of new compounds	S4
EPR data of complex **1**–**4**	S9
Additional computational results	S10
Titration data for stability constant determinations	S14
Additional CV data in water	S15
UV-Vis data in the presence of water	S17
Controlled potential electrolysis and oxygen evolution	S19
Additional Mass Spectrometry Data	S20

Table S1. Summary of crystallographic data for complexes **1–4**.

	Complex **1**	Complex **2**	Complex **3**	Complex **4**
CCDC	2178876	2178877	2178878	2301428
Crystal data				
Chemical formula	$C_{51}H_{53}Cl_6Mn_3N_{15}O_{31}$	$C_{19}H_{15}F_6MnN_5O_7S_2$	$C_{19}H_{18}Cl_2MnN_6O$	$C_{21}H_{18}F_6MnN_6O_7S_2$
M_r	1746.58	658.42	472.23	699.47
Crystal system, space group	Triclinic, *P*1	Monoclinic, $P2_1/c$	Monoclinic, *C*2/*c*	Orthorhombic, *Pbca*
Temperature (K)	100	200	160	100
a, *b*, *c* (Å)	11.820 (1), 12.7584 (11), 13.3067 (13)	8.0555 (2), 10.3178 (2), 29.9387 (6)	19.775 (5), 17.494 (4), 14.574 (4)	15.1833 (6), 13.4972 (4), 24.4882 (9)
α, β, γ (°)	109.691 (6), 107.464 (5), 99.058 (5)	90, 93.604 (1), 90	90, 129.016 (8), 90	90, 90, 90
V (Å^3)	1726.0 (3)	2483.44 (9)	3917.3 (17)	5018.4 (3)
Z	1	4	8	8
μ (mm^{-1})	7.41	6.80	8.20	6.79
Crystal size (mm)	0.23 × 0.14 × 0.10	0.30 × 0.09 × 0.03	0.33 × 0.23 × 0.12	0.29 × 0.13 × 0.10
Data collection				
T_{min}, T_{max}	0.276, 0.519	0.236, 0.832	0.176, 0.437	0.243, 0.547
No. of measured, independent and observed [$I > 2\sigma(I)$] reflections	45330, 11582, 10166	37643, 5345, 4317	25587, 4230, 3698	46443, 5382, 4062
R_{int}	0.072	0.076	0.048	0.173
$(\sin \theta/\lambda)_{max}$ (Å^{-1})	0.639	0.638	0.639	0.638
Refinement				
$R[F^2 > 2\sigma(F^2)]$, $wR(F^2)$, S	0.069, 0.175, 1.07	0.052, 0.107, 1.09	0.037, 0.093, 1.04	0.082, 0.191, 1.18
No. of reflections	11220	5065	4230	4579
No. of parameters	965	400	266	394
No. of restraints	3	138	0	110
H-atom treatment	H-atom parameters constrained	H-atom parameters constrained	H-atom parameters constrained	H atoms treated by a mixture of independent and constrained refinement
Weighting Scheme	$w = 1/[\sigma^2(F_o^2) + (0.0710P)^2 + 5.8218P]$ where $P = (F_o^2 + 2F_c^2)/3$	$w = 1/[\sigma^2(F_o^2) + (0.0378P)^2 + 10.6021P]$ where $P = (F_o^2 + 2F_c^2)/3$	$w = 1/[\sigma^2(F_o^2) + (0.0414P)^2 + 8.472P]$ where $P = (F_o^2 + 2F_c^2)/3$	$w = 1/[\sigma^2(F_o^2) + (0.0618P)^2 + 42.8721P]$ where $P = (F_o^2 + 2F_c^2)/3$
$\Delta\rho_{max}$, $\Delta\rho_{min}$ (e Å^{-3})	0.885, −0.694	0.61, −0.442	0.83, −0.29	1.06, −0.55

Table S2. Selected bond lengths (Å) and angles (°) for complex **1–4**. Numbering scheme is as given in Figure 4.

	**1**[a]	**1'**	**1''**	**2**	**3**	**4**
d (Mn–$N1_{bpy}$)	2.175(8)	2.186(9)	2.216(8)	2.183(3)	2.273(2)	2.266(5)
d (Mn–$N2_{bpy}$)	2.269(8)	2.241(8)	2.246(8)	2.248(3)	2.356(2)	2.275(5)
d (Mn–$N3_{imi}$)	2.131(8)	2.158(10)	2.127(9)	2.151(3)	2.331(2)	2.242(5)
d (Mn–$N4_{imi}$)	N/A	N/A	N/A	N/A	2.221(2)	2.233(5)
d (Mn–O/Cl_1)	2.186(11)[b]	2.191(9)[b]	2.211(10)[b]	2.253(3)[d]	2.4855(9)[e]	2.204(4)[d]
d (Mn–O/Cl_2)	2.211(9)[b]	2.291(9)[c]	2.183(13)[c]	2.258(2)[d]	2.5414(8)[e]	2.256(5)[d]
∠N1–Mn–N2	75.1(3)	76.4(3)	75.5(3)	76.34(10)	69.74(7)	72.06(18)
∠N2–Mn–N3	85.7 (3)	86.9(3)	86.0(3)	86.11(10)	75.93(7)	80.47(19)
∠N3–Mn–N4	N/A	N/A	N/A	N/A	78.93(8)	83.77(19)

[a]Unit cell contains three inequivalent molecules of compound; **1** is ligated by two water molecules (in addition to BPI1 and an acetonitrile molecule) while in **1'** and **1''** one of the water molecules is replaced by a perchlorate ligand. [b]Distance to OH_2 ligand(s) [c]Distance to perchlorate ligand. [d] Distance to triflate ligands [e]Distance to chloride ligands.

Table S3. Angular distortion parameters[a]

	**1**	**2**	**3**	**4**	**(bpy^{NHEt}PY2Me)Mn(OTf)$_2$[b]**
Σ (°)	70.47	44.61	186.02	189.21	114.38
Θ (°)	239.7	171.50	1193.81	1997.63	392.38

[a]Calculated using OctaDist [49] [b] From reference [58]

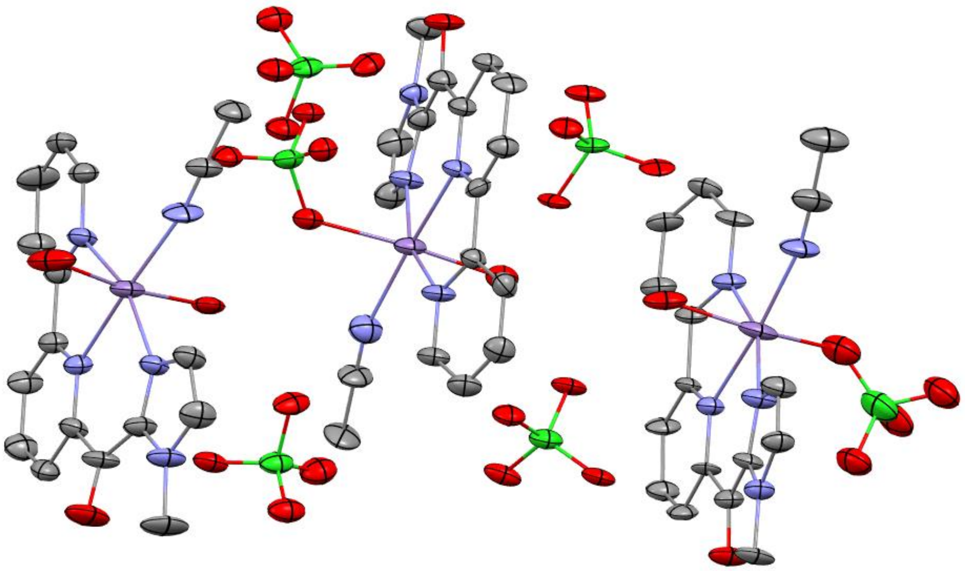

Figure S1. Complete asymmetric unit for complex **1**, showing three crystallographically independent cations. Hydrogen atoms and disorder components omitted for clarity and thermal ellipsoids shown at 50% probability.

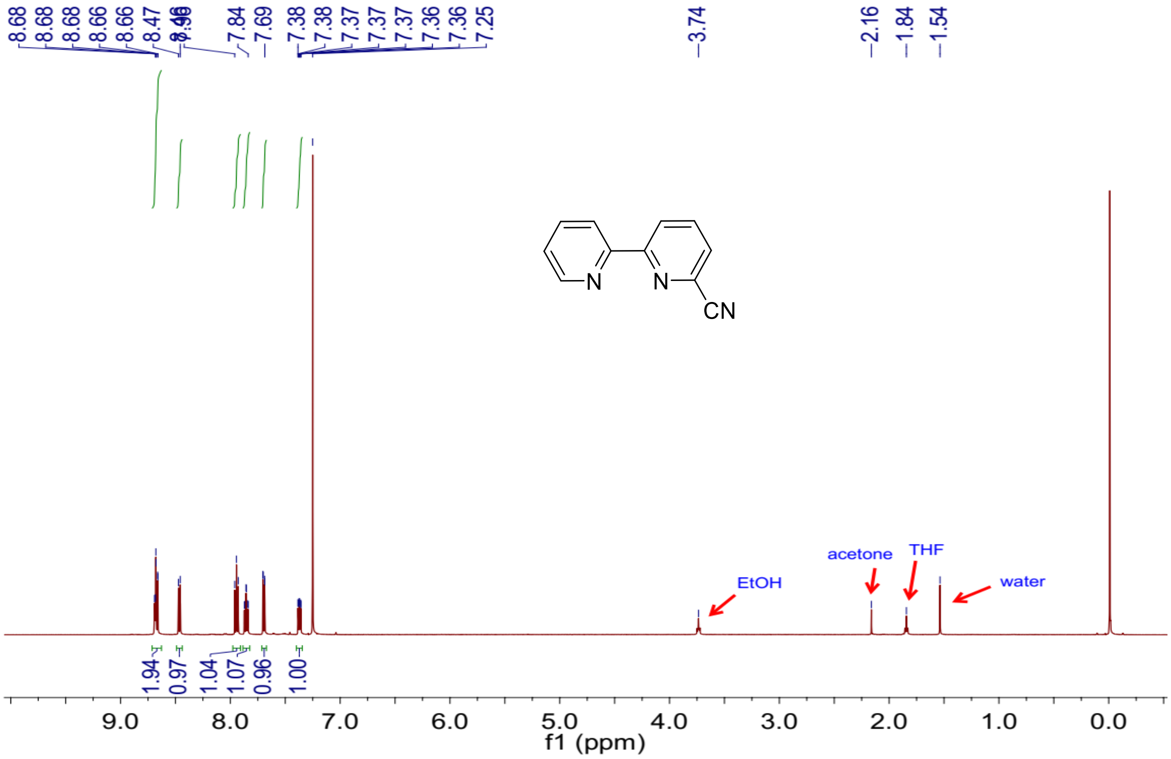

Figure S2. Room-temperature ^{1}H NMR spectrum of 6-cyano-2,2'-bipyridine, recorded at 500 MHz in $CDCl_3$.

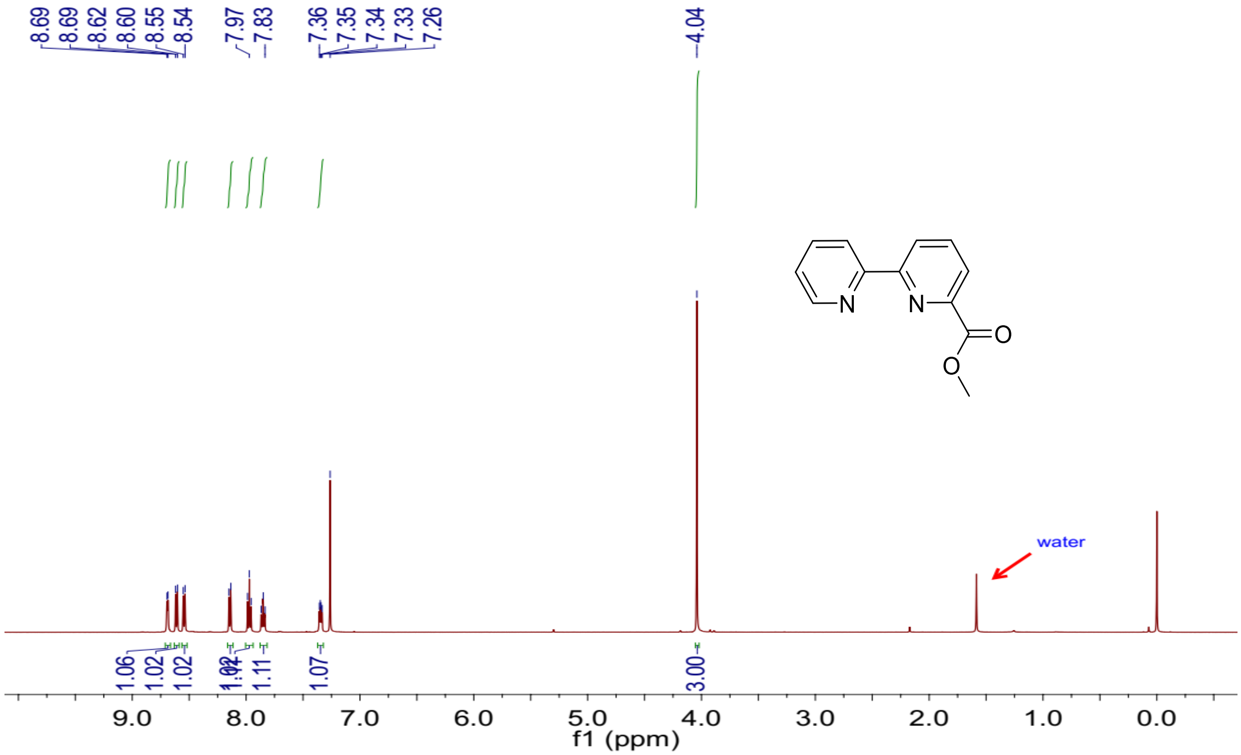

Figure S3. Room-temperature ^{1}H NMR spectrum of 6-methoxycarbonyl-2,2'-bipyridine, recorded at 500 MHz in $CDCl_3$.

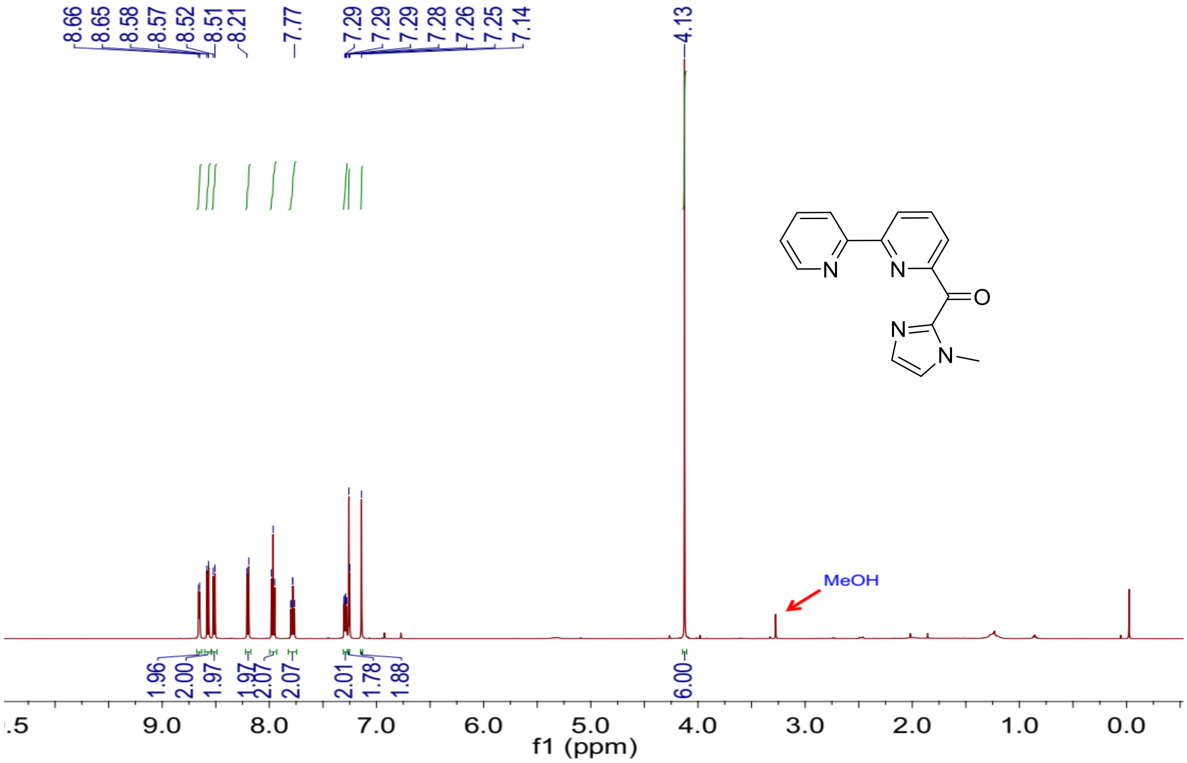

Figure S4. Room-temperature ^{1}H NMR spectrum of BPI1, recorded at 500 MHz in $CDCl_3$.

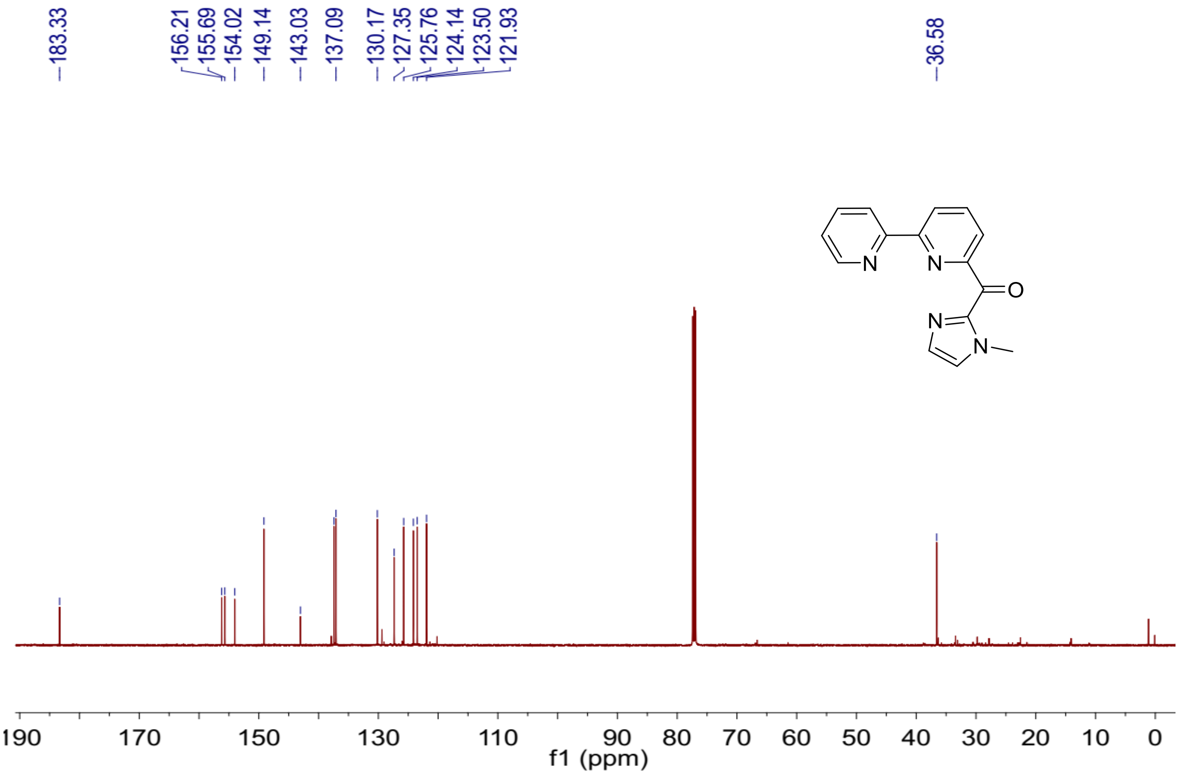

Figure S5. Room-temperature $^{13}C\{^{1}H\}$ NMR spectrum of BPI1, recorded at 126 MHz in $CDCl_3$.

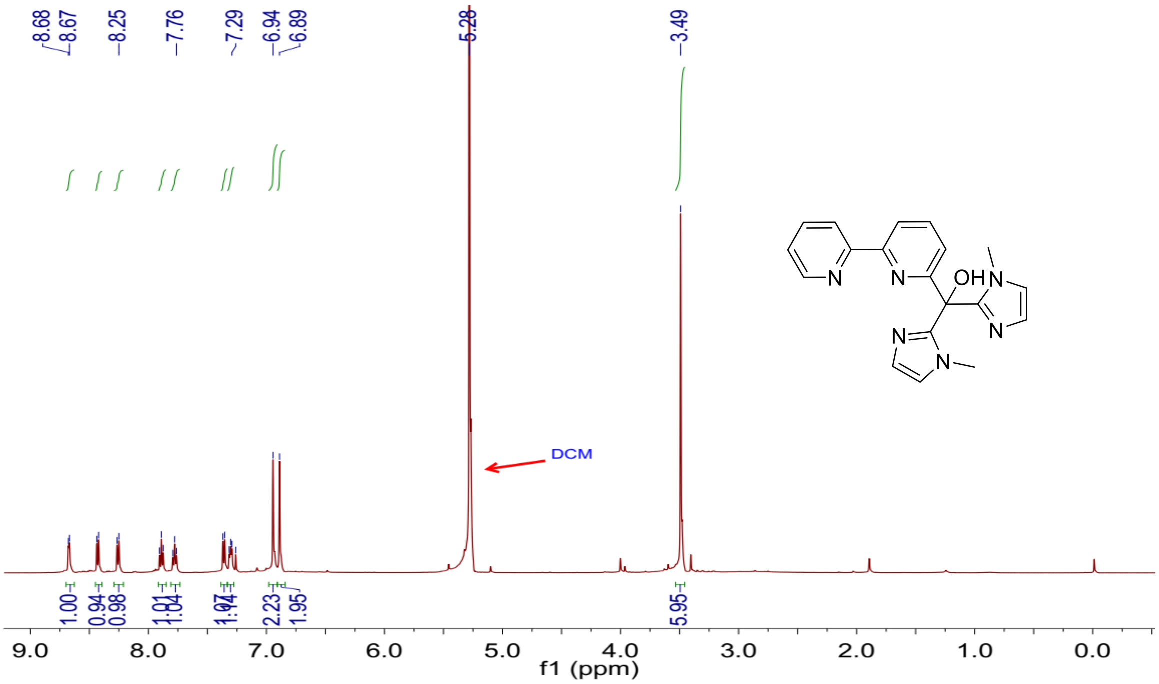

Figure S6. Room-temperature ¹H NMR spectrum of BPI2, recorded at 500 MHz in $CDCl_3$.

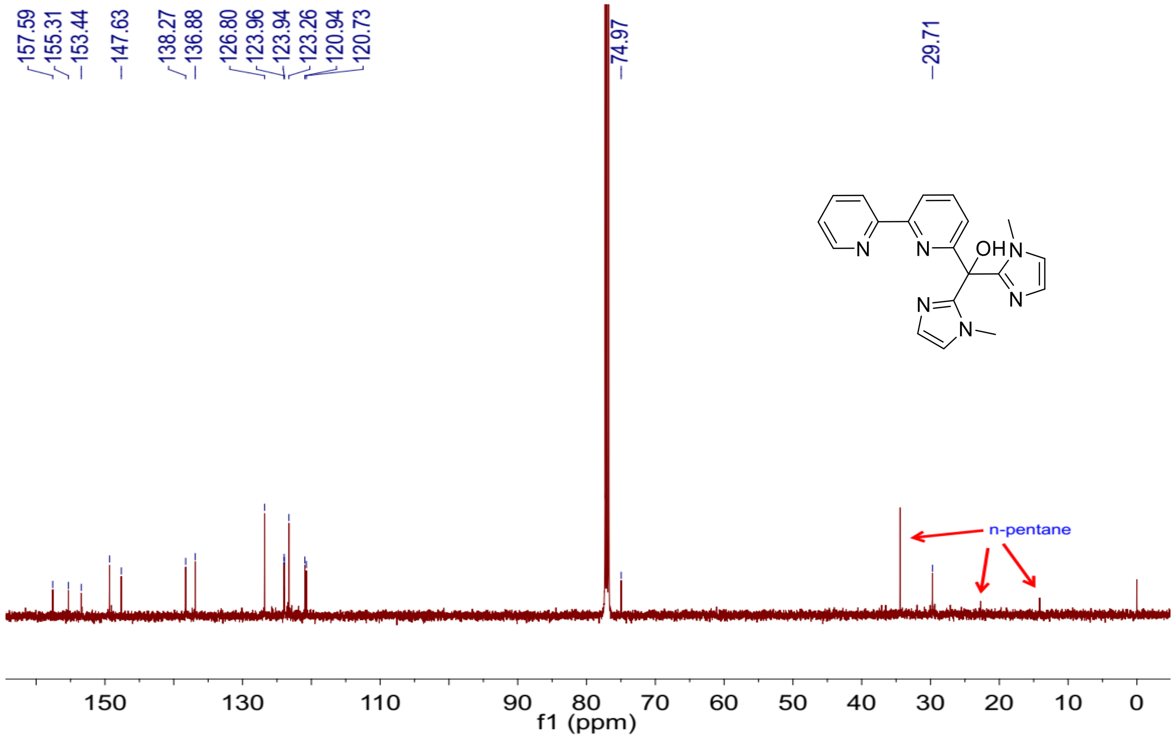

Figure S7. Room-temperature ¹³C{¹H} NMR spectrum of BPI2, recorded at 126 MHz in $CDCl_3$.

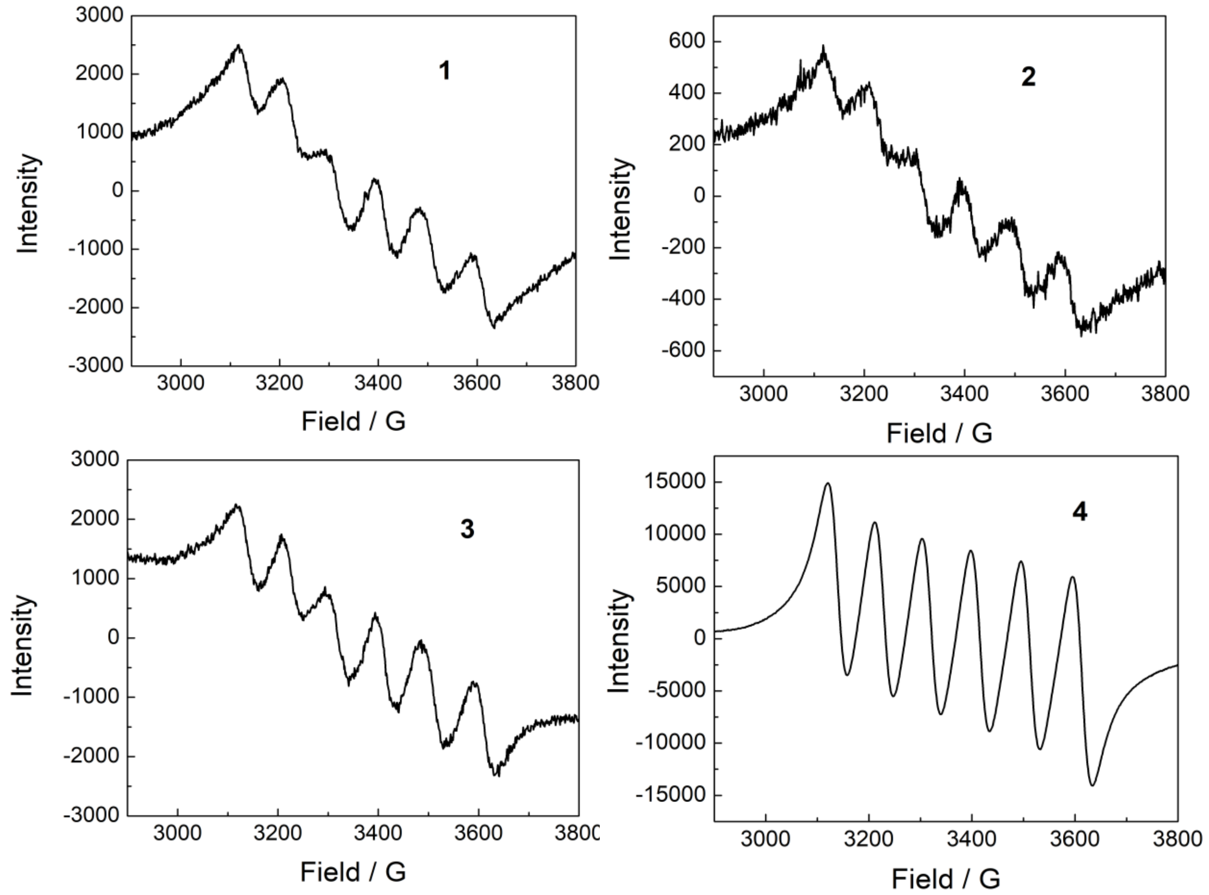

Figure S8. X-band EPR spectra of complex **1**–**4** (0.5 mM), recorded at room temperature in a solution mixure (EtOH:MeOH=4:1).

Complex 1

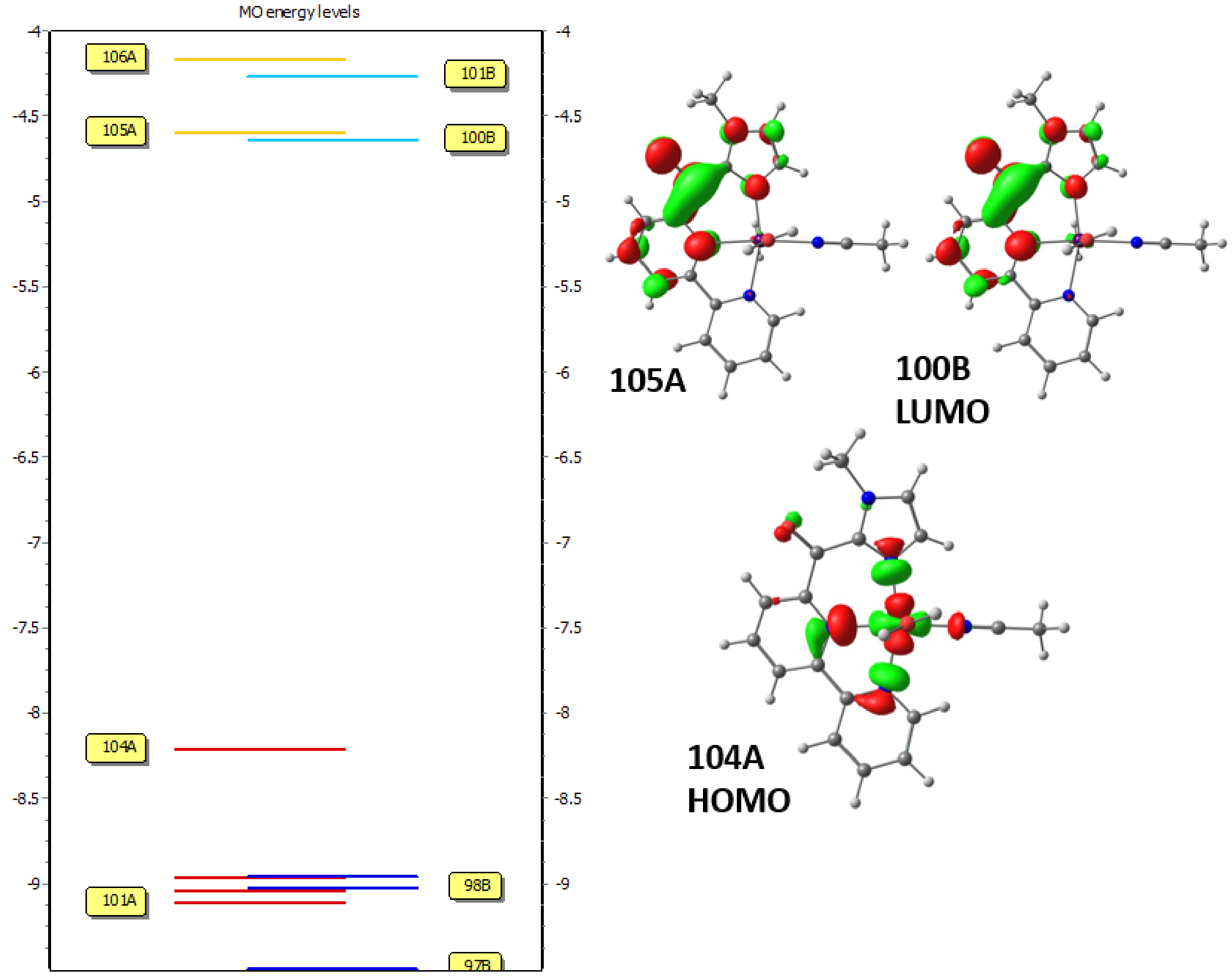

Figure S9. Orbital energy diagram (energies given in eV) and depictions of the Kohn-Sham frontier orbitals for complex 1 with two ancillary water ligands (orbital isosurfaces shown at 0.05).

Complex 3

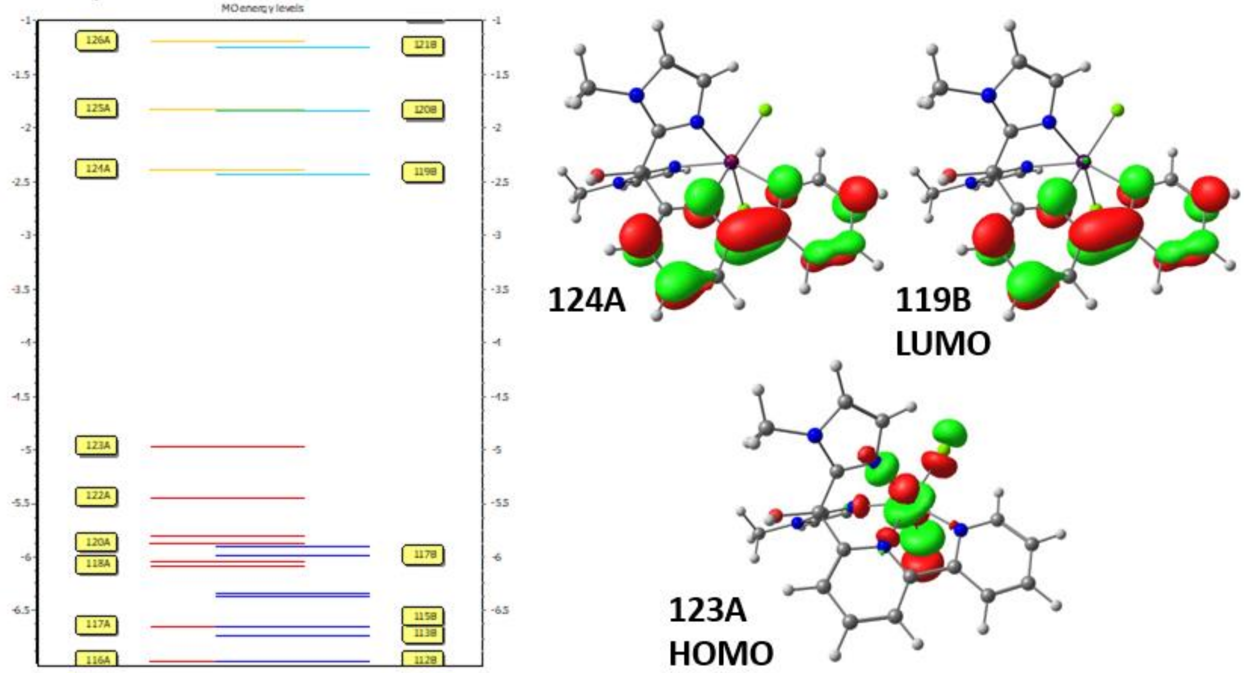

Figure S10. Orbital energy diagram (energies given in eV) and depictions of the Kohn-Sham frontier orbitals for complex 3 (orbital isosurfaces shown at 0.05).

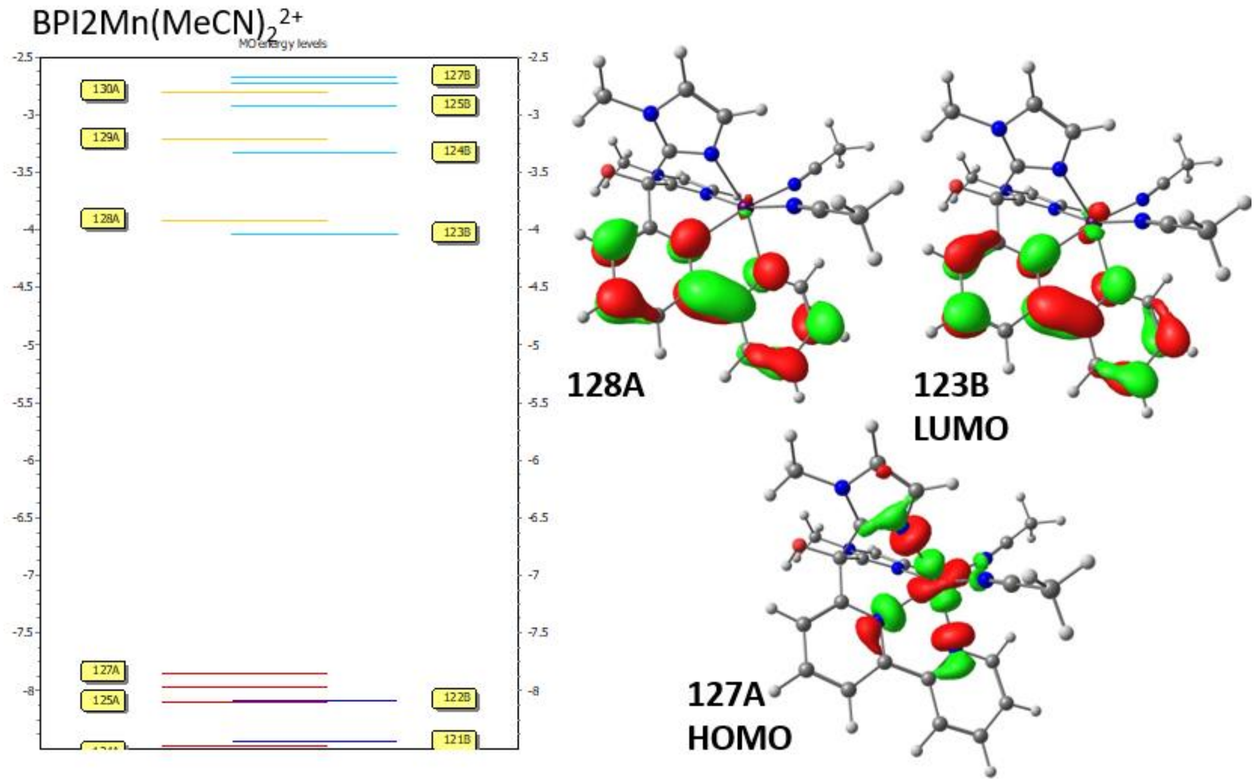

Figure S11. Orbital energy diagram (energies given in eV) and depictions of the Kohn-Sham frontier orbitals for the BPI2Mn(MeCN)$_2^{2+}$ cation (orbital isosurfaces shown at 0.05).

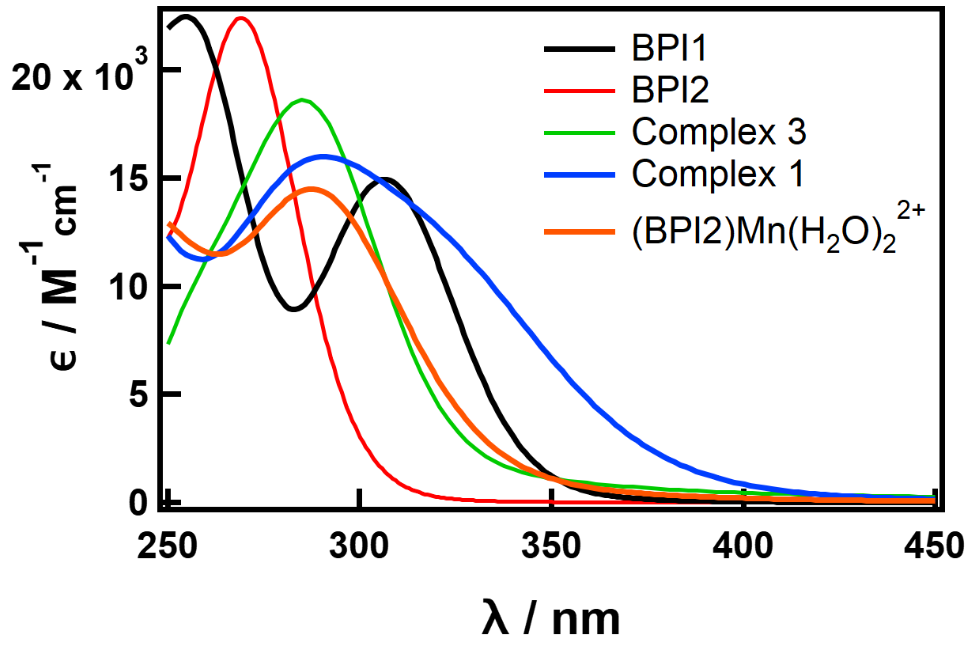

Figure S12. Overlay of calculated spectra (TD-DFT) for ligands and selected complexes. Gaussian broadening of 0.28 eV (HWHM) was applied to generate the simulated spectra.

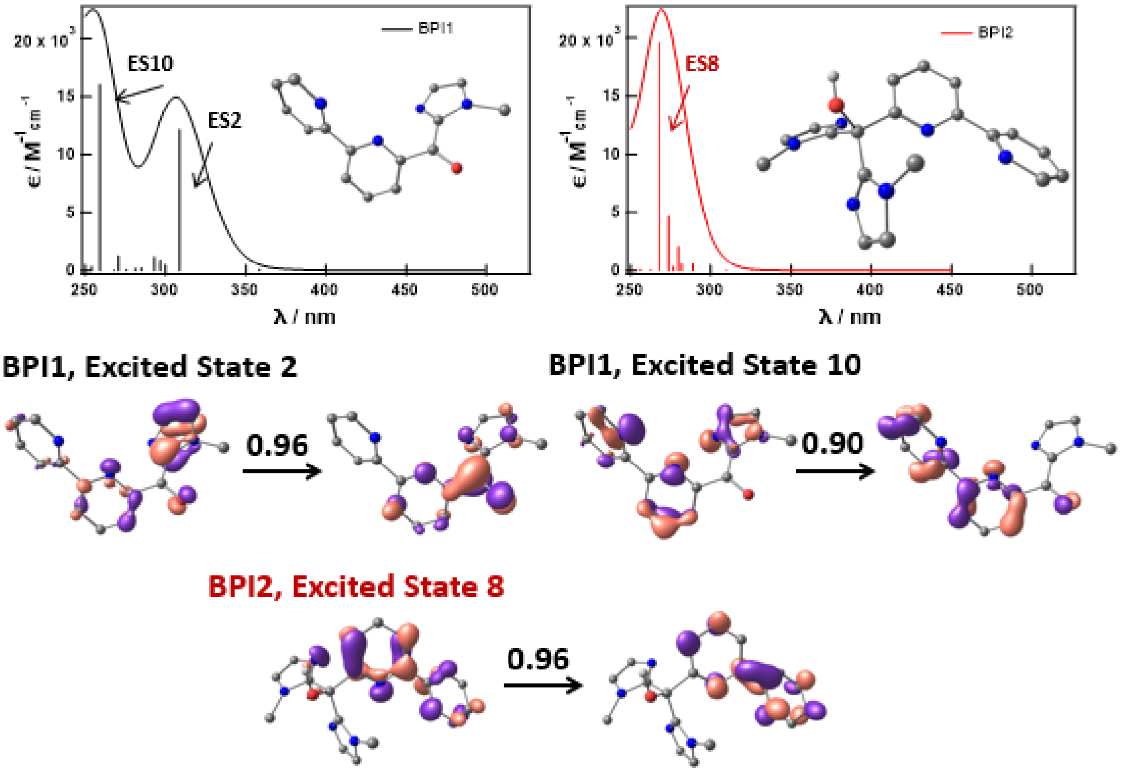

Figure S13. Calculated spectra and selected natural transition orbitals (NTOs) for the ligands BPI1 and BPI2. Optimized structures are shown as insets; hydrogen atoms attached to carbon are omitted. Transitions between the dominant NTOs for selected excited states are shown on the bottom; numbers above the arrows indicate the occupancy of the corresponding NTOs, which is indicative of their relative contribution to the transition. Orbital contours are shown at an isovalue of 0.06.

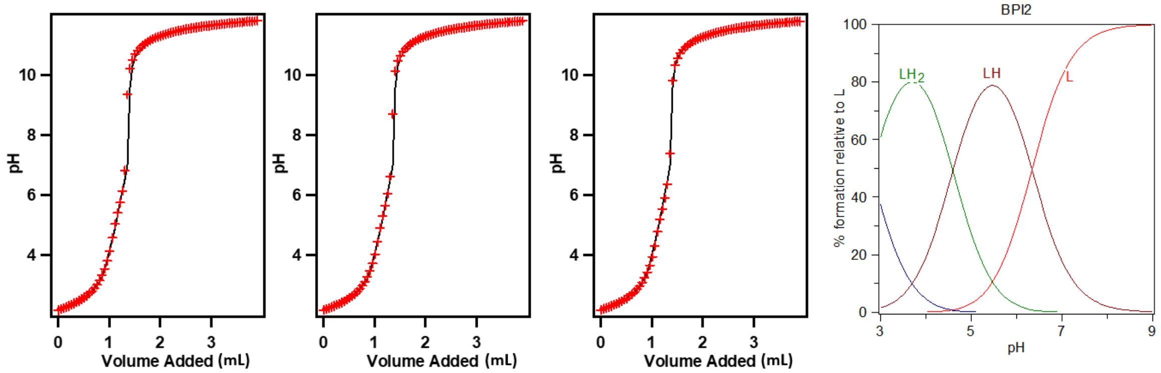

Figure S14. Titrations of BPI2 ligand to determine protonation constants (see Table 2 in main text). Three independent trials are shown and a simulated speciation diagram is shown on the right (ligand concentration for simulation = 1 mM). Titration experiments were carried out on a 6 mL aqueous solution of approximately 1.7 mM ligand in 11.6 mM HCl, with 0.15 M NaCl to maintain constant ionic strength. The titrant was 0.496 M aqueous NaOH.

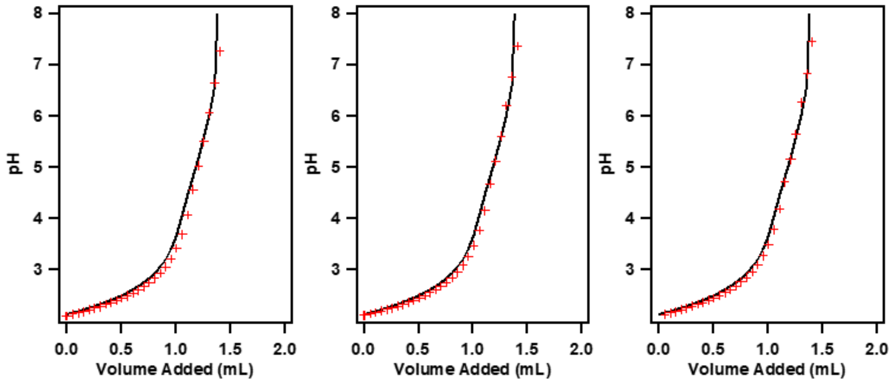

Figure S15. Titrations of BPI2 in the presence of 1 equiv of Mn^{2+} in order to determine the manganese association constant (K_{Mn}, see Table 1 in main text). Three independent trials are shown and simulated speciation diagrams at 2 different concentrations (ligand concentration = Mn^{2+} concentration). Titration experiments were carried out on a 6 mL aqueous solution of 1.47 mM complex **4** in 11.6 mM HCl, with 0.15 M NaCl to maintain constant ionic strength. The titrant was 0.496 M aqueous NaOH. In fitting the data, ligand protonation constants were fixed to independently determined values (see Figure S11 and Table 1 in main text) and only K_{Mn} was allowed to vary.

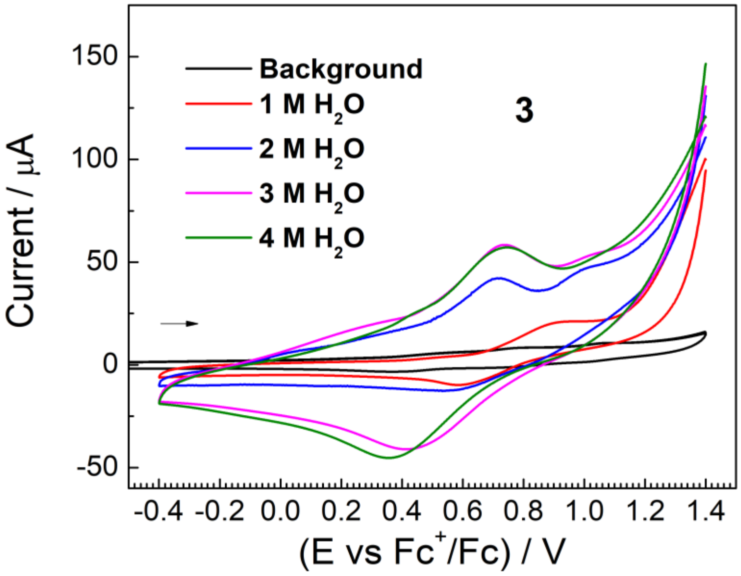

Figure S16. Overlaid cyclic voltammograms of complex **3** (0.24 mM) with increasing amounts of H_2O in acetonitrile with 0.1 M NBu_4PF_6 supporting electrolyte, using a glassy carbon working electrode and a scan rate of 100 mV/s. The arrow indicates the scan direction. Background scan contains no manganese complex and no water.

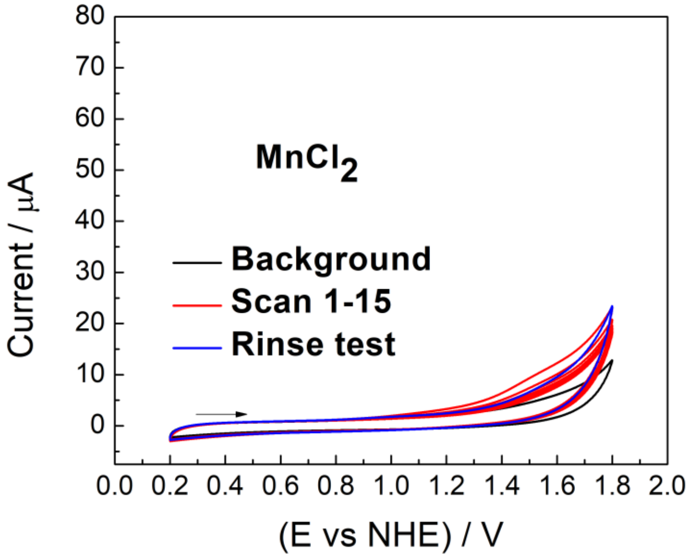

Figure S17. Consecutive cyclic voltammograms of $MnCl_2$ (0.24 mM) at pH = 7. The data were recorded in 0.1 M phosphate buffer, using a glassy carbon working electrode and a scan rate of 100 mV/s. The arrow indicates the scan direction.

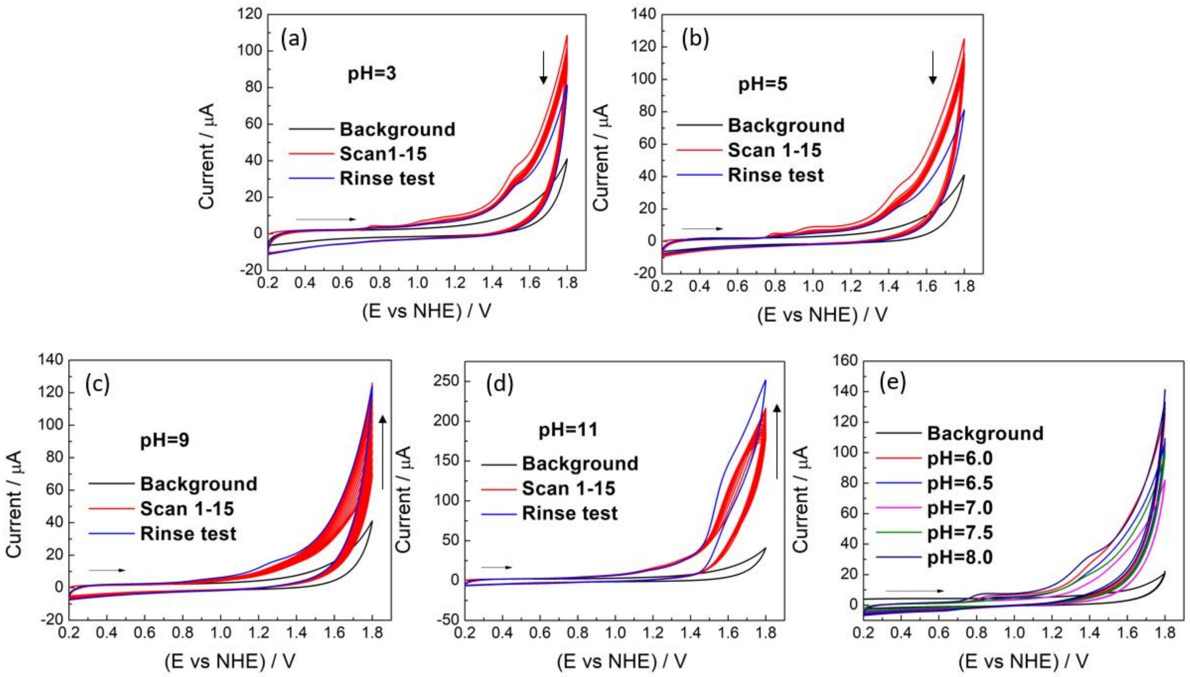

Figure S18. Consecutive cyclic voltammograms of complex **4** (0.24 mM) at varying pHs: (a) pH = 3, (b) pH = 5, (c) pH = 9, (d) pH = 11 and (e) pH = 6.0–8.0. The data were recorded in 0.1 M phosphate buffer, using a glassy carbon working electrode and a scan rate of 100 mV/s. The horizontal arrows indicate the scan direction and the vertical arrows indicate the direction of change in current upon subsequent scans.

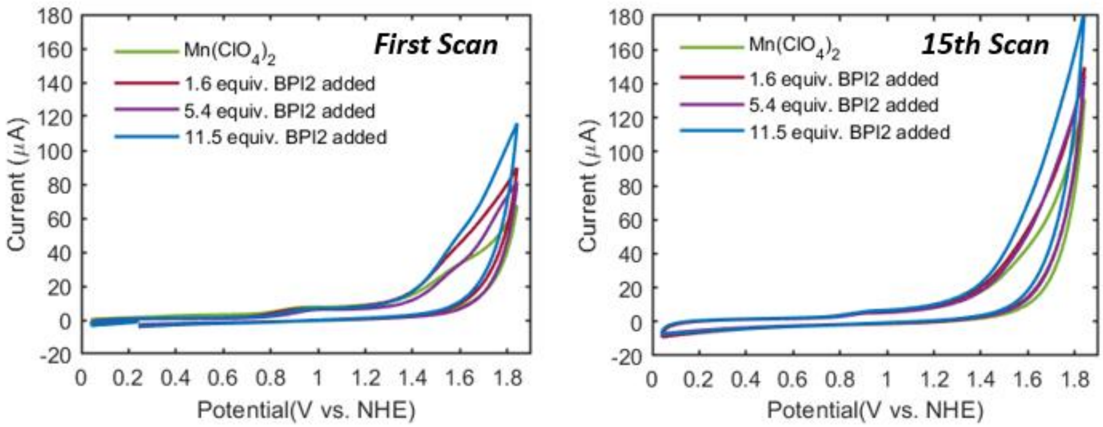

Figure S19. Aqueous (0.1 M phosphate buffer, pH 7) cyclic voltammetry scans of 0.24 mM $Mn(ClO)_4)_2$ in the presence of added BPI2 ligand. Each solution was scanned 15 fifteen times and the overlaid data for the first (left) and last (right) scans are shown.

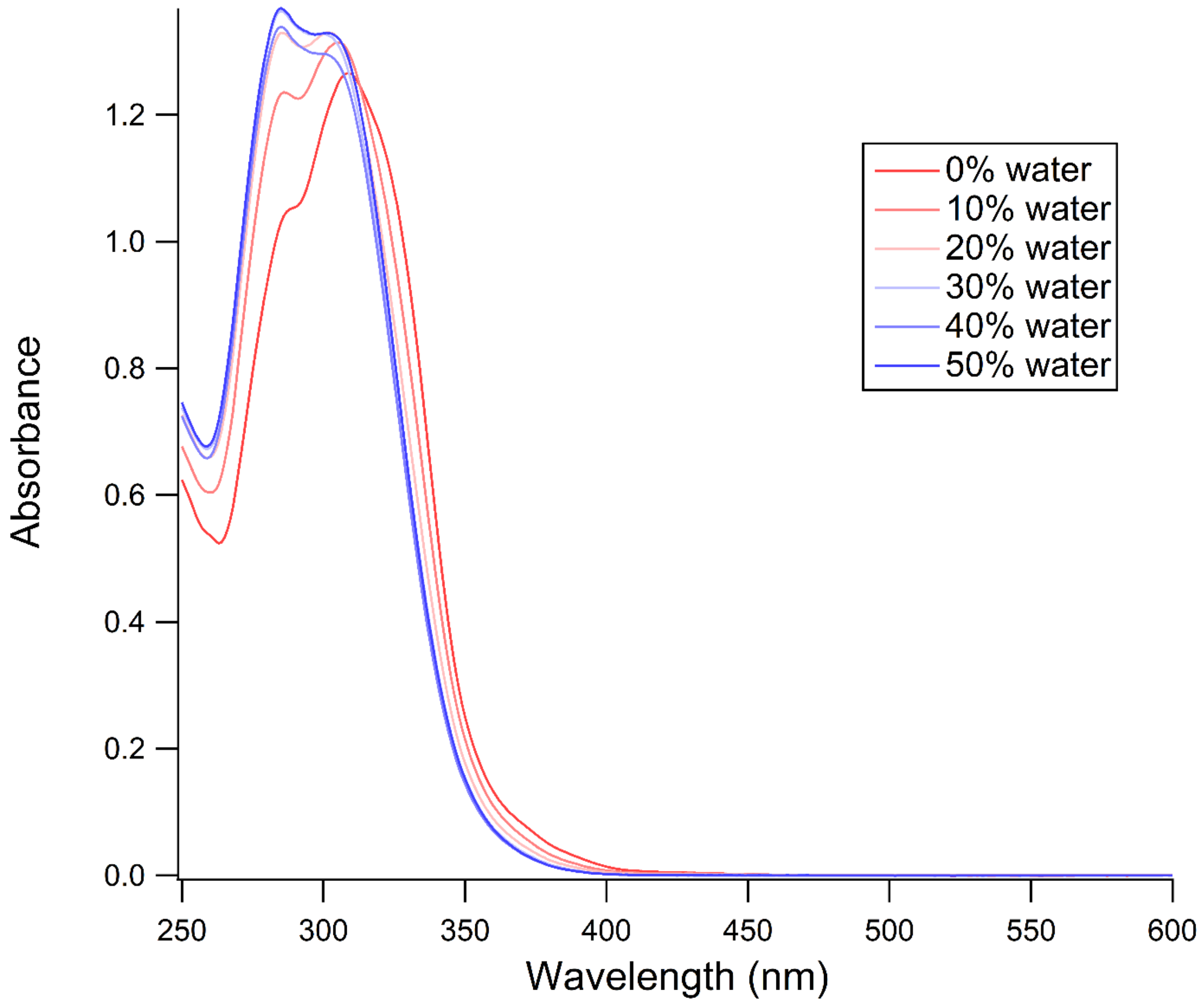

Figure S20. UV-Vis spectra of complex **1** (0.1 mM) in acetonitrile/water mixtures with increasing amounts of water.

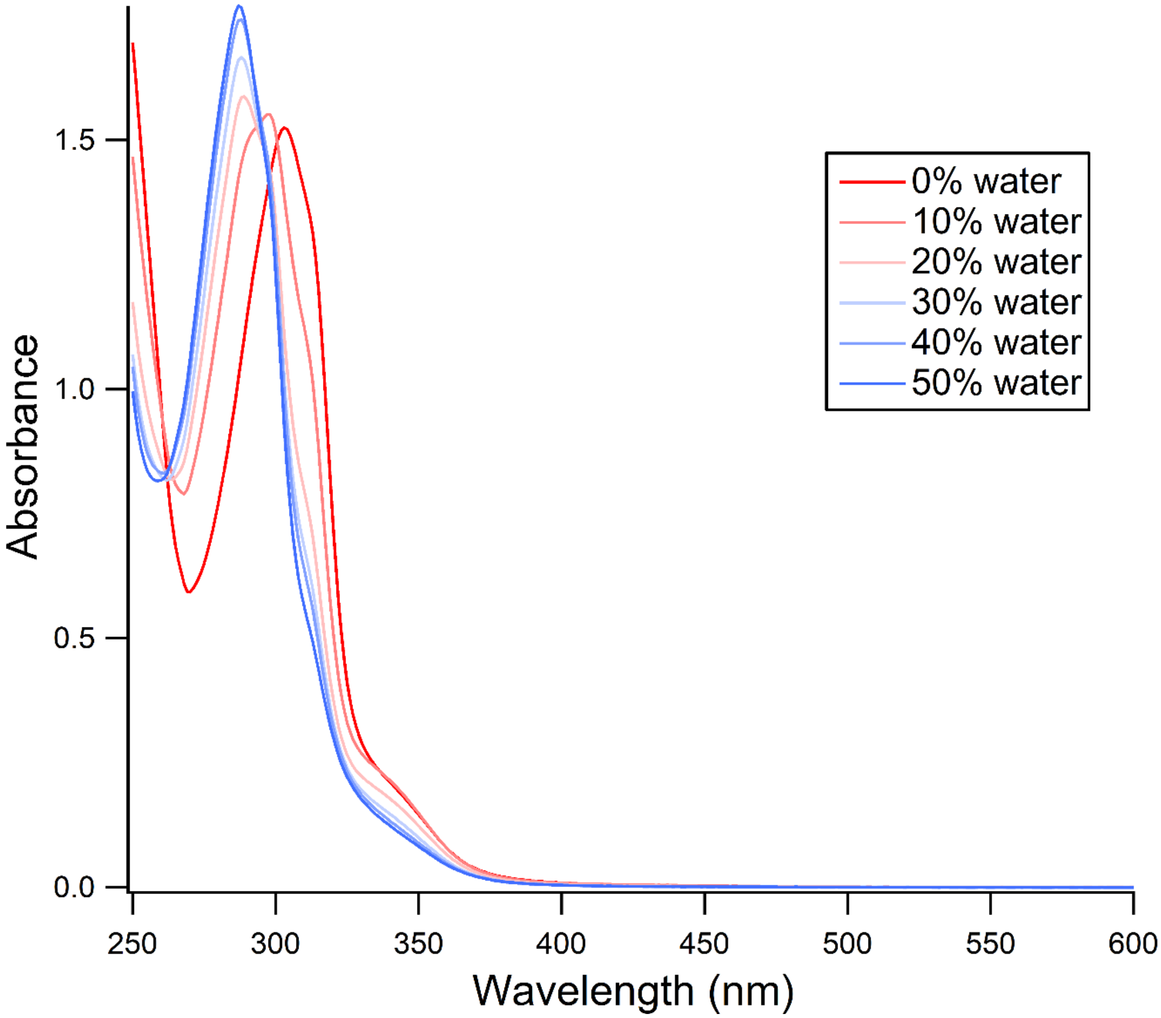

Figure S21. UV-Vis spectra of complex **4** (0.1 mM) in acetonitrile/water mixtures with increasing amounts of water.

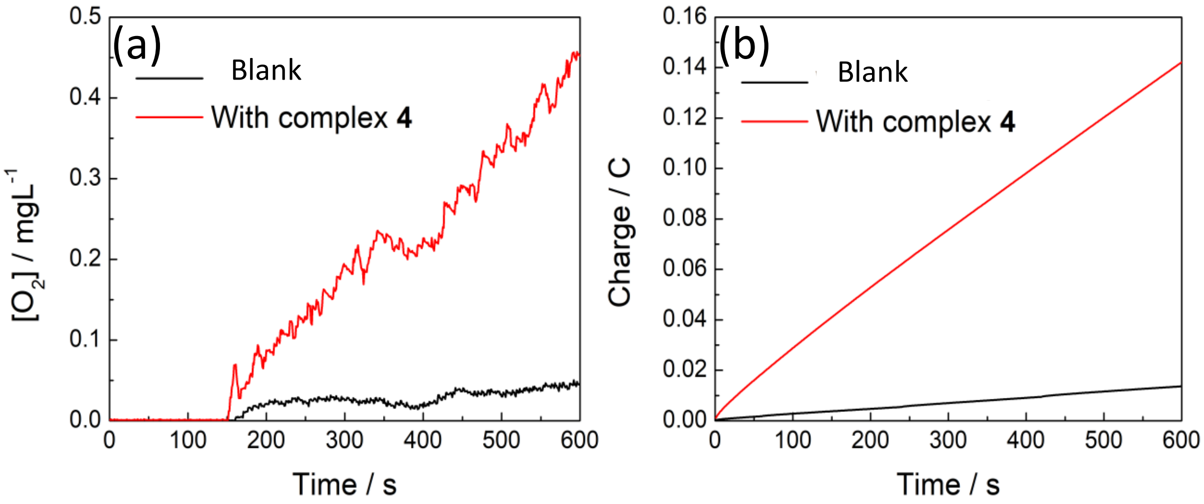

Figure S22. (a) Oxygen evolution and (b) charge during bulk electrolysis at the applied potential of 1.60 V vs NHE. Conditions: 0.143 mM complex **4**; 0.1 M phosphate buffer solution at pH 7.0; Square-plate glassy carbon electrode (8.0 cm^2). Background scan was taken under identical conditions in the absence of complex. O_2 concentration before 150 seconds is below the probe detection limit.

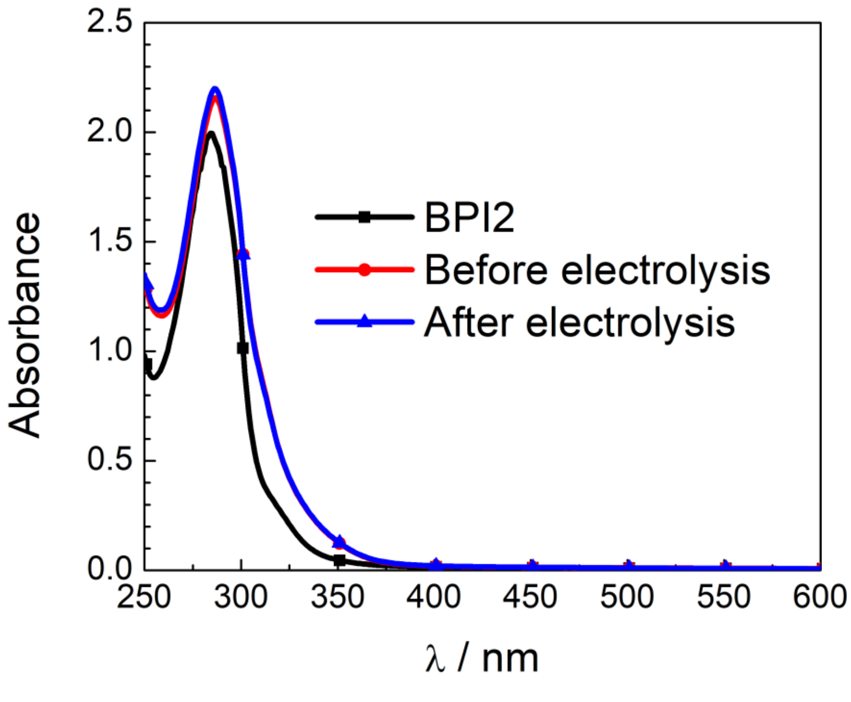

Figure S23. Overlaid electronic absorption spectra of complex **4** before and after bulk electrolysis for 20 min at the applied potential of 1.6 V vs NHE. Condition: 0.143 mM complex **4**; 0.1 M phosphate buffer solution at pH 7.0; Square-plate glassy carbon electrode (8.0 cm^2). Data was collected in intervals of 1 nm, and symbols are included on each plot are to help distinguish the overlaid spectra.

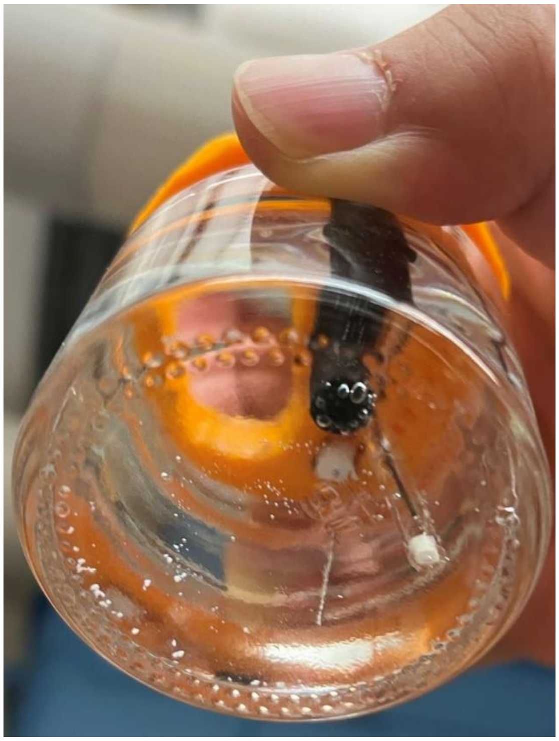

Figure S24. Oxygen formation during bulk electrolysis at the applied potential of 1.60 V vs NHE.

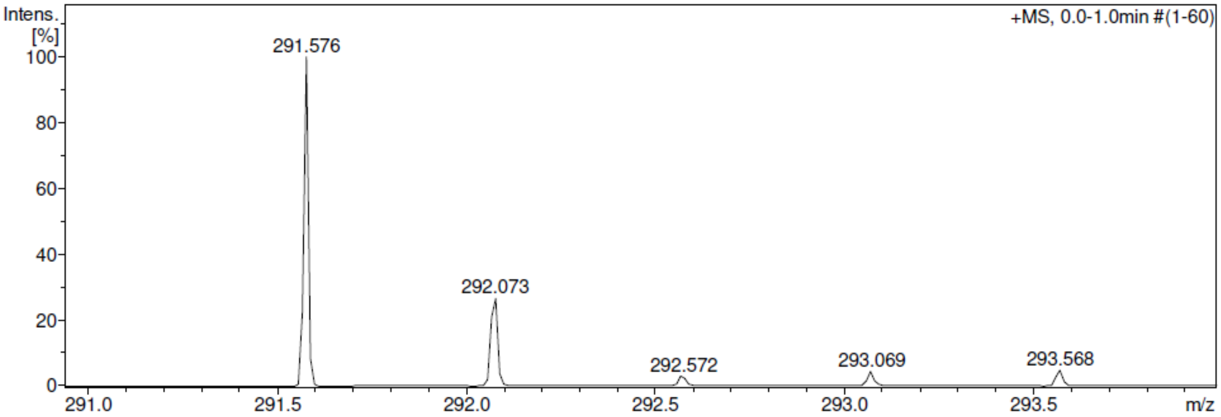

Figure S25. Expanded view of the peak at m/z = 291.57 amu in the mass spectrum of complex 1
